# Haem Iron Intake Is Associated with Increased Major Adverse Cardiovascular Events, All-Cause Mortality, Congestive Cardiac Failure, and Coronary Revascularisation in Older Men: The Concord Health and Ageing in Men Project

**DOI:** 10.1007/s12603-023-1945-6

**Published:** 2024-01-04

**Authors:** Rebecca Luong, R.V. Ribeiro, A. Rangan, V. Naganathan, F. Blyth, L.M. Waite, D.J. Handelsman, D.G. Le Couteur, M.J. Seibel, V. Hirani

**Affiliations:** 1 Nutrition and Dietetics Group, Sydney Nursing School, Faculty of Medicine and Health, The University of Sydney, Sydney, NSW, Australia; 2 ARC Centre of Excellence in Population Ageing Research (CEPAR), The University of Sydney, Sydney, NSW, Australia; 3 Level 4, Charles Perkins Centre D17, University of Sydney, 2006, Sydney, NSW, Australia; 4 School of Life and Environmental Sciences, Faculty of Science, The University of Sydney, Sydney, NSW, Australia; 5 Centre for Education and Research on Ageing, Concord Hospital, The University of Sydney, Concord, NSW, Australia; 6 Concord Clinical School, Faculty of Medicine and Health, The University of Sydney, Concord, NSW, Australia; 7 School of Public Health, The University of Sydney, Sydney, NSW, Australia; 8 ANZAC Research Institute, The University of Sydney and Concord Hospital, Concord, NSW, Australia; 9 Andrology Department, Concord Hospital, Concord, NSW, Australia

**Keywords:** Dietary iron, old men, mortality, coronary artery disease, heart failure

## Abstract

**Background:**

Nutritional intake can influence major adverse cardiovascular events (MACE). Dietary iron is found in two forms: haem-iron (HI) only found in animal sources and non-haem iron (NHI) present mostly in plant sources.

**Objective:**

We evaluated the associations between dietary iron intakes with MACE and iron status biomarkers.

**Design:**

Prospective cohort study.

**Setting:**

The Concord Health and Ageing in Men Project, Sydney, Australia.

**Participants:**

539 community-dwelling older Australian men aged 75 years and older.

**Methods:**

Men underwent nutritional assessment using a validated diet history questionnaire. Entries were converted to food groups and nutrients. The dietary calculation was used to derive HI and NHI intakes from total iron intakes. Analyses of iron intakes with iron status biomarkers were conducted using linear regression, and with MACE and individual endpoints were conducted using Cox regression. Five-point MACE comprised of all-cause mortality, myocardial infarction (MI), congestive cardiac failure (CCF), coronary revascularisation, and/or ischaemic stroke. Four-point MACE included the four endpoints of MI, CCF, coronary revascularisation, and/or ischaemic stroke, and excluded all-cause mortality.

**Results:**

At a median of 5.3 (4.6–6.3) years follow-up, the incidences were: 31.2% (n = 168) five-point MACE, 17.8% (n = 96) four-point MACE excluding all-cause mortality, 20.1% (n = 111) all-cause mortality, 11.3% (n = 61) CCF, and 3.1% (n = 15) coronary revascularisation. In adjusted analyses, higher HI intake (per 1mg increment) was associated with increased five-point MACE (HR: 1.45 [95% CI: 1.16, 1.80, P =.001]), four-point MACE excluding all-cause mortality (HR: 1.64 [95% CI: 1.26, 2.15, P <.001]), all-cause mortality (HR: 1.51 [95% CI: 1.15, 1.99, P =.003]), CCF (HR: 2.08 [95% CI: 1.45, 2.98, P <.001]), and coronary revascularisation (HR: 1.89 [95% CI: 1.15, 3.10, P =.012]). Compared with the bottom tertile of NHI intake, the middle tertile of NHI intake was associated with reduced risk of all-cause mortality (HR: 0.56 [95% CI: 0.33, 0.96, P =.035]). Total iron intake was not associated with MACE and individual endpoints. Dietary iron intakes were not associated with serum iron and haemoglobin.

**Conclusion:**

Higher haem iron intake was independently associated with increased risks of five-point MACE, four-point MACE excluding all-cause mortality, all-cause mortality, CCF, and coronary revascularisation in older men over 5 years.

## Introduction

Ageing increases the risk of cardiovascular disease (CVD) development which is the leading cause of death worldwide (1). Older adults are also susceptible to poor nutritional intake due to medical, physiological, and social changes (2). Older men are at higher risk of nutritional inadequacy due to limited meal preparation skills (3), and also have double the rates of CVD than older women (1). There is emerging evidence that diet has different associations with CVD in older adults compared with younger age groups, with some research demonstrating weaker or no associations (4, 5).

Although there has been a shift towards food-based dietary recommendations to meet nutritional requirements for prevention of deficiency and diseases (6), it is important to investigate nutrients present in foods that could influence health outcomes. Iron is found in food in two forms: haem iron (HI) and non-haem iron (NHI) (7). HI is only found in animal sources and NHI is present mostly in plant sources (7). HI intake, but not total iron and NHI intakes, is associated with increased risk of diabetes in adults, a major risk factor for CVD (8). Dietary iron intakes also show variable cross-sectional associations with components of the metabolic syndrome in adults (9). Hence, the consumption of different types of dietary iron may have different associations with major adverse cardiovascular events (MACE).

Studies relating dietary iron intakes and CVD have mostly been conducted in adults aged less than 75 years (7, 10, 11). Furthermore, previous studies mostly involved CHD and CVD as the outcome rather than MACE, and the individual endpoints of congestive cardiac failure (CCF) and coronary revascularisation have not been examined (7, 10). The associations between dietary iron intakes with MACE and the individual endpoints in older men aged 75 years and over have not been investigated. Iron status biomarkers which can be affected by inflammation often presented in ageing or chronic disease, have also not been consistently associated with dietary iron intakes in older adults (12, 13, 14).

The aim of this study was to evaluate the associations between dietary iron intakes (total iron, HI and NHI) with MACE and individual endpoints of MACE in older men aged 75 years and over through prospective analyses. A secondary aim was to examine cross-sectional associations between dietary iron intakes with iron status biomarkers in this same cohort.

## Methods

### Study Participants

**Figure 1:**
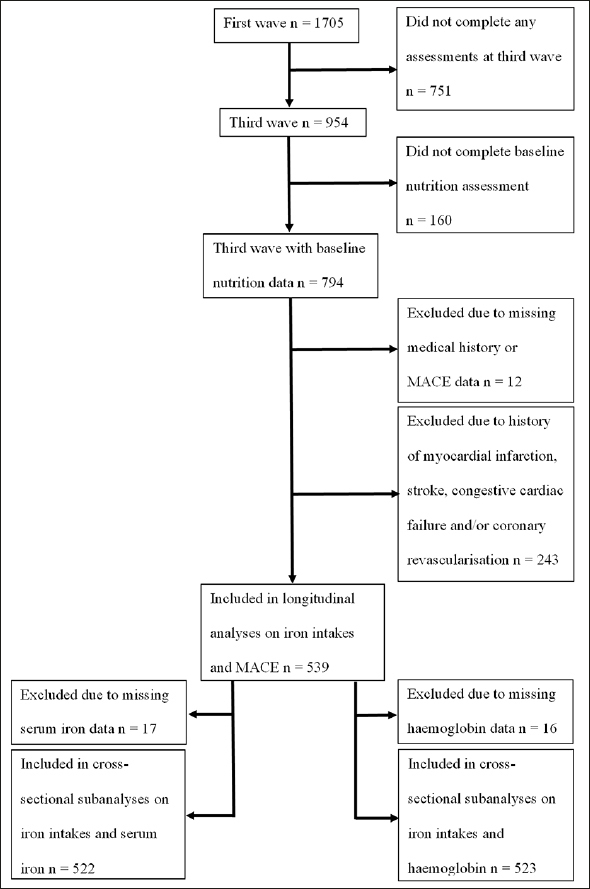
Flow diagram of participants included in longitudinal analyses and cross-sectional sub analyses

The Concord Health and Ageing in Men Project (CHAMP) is a prospective cohort study of ageing in men. A total of 1,705 community-dwelling men aged 70 years and over, registered as living in a defined geographical region (the Local Government Areas of Burwood, Canada Bay and Strathfield) from the compulsory Electoral Roll, were recruited in the first wave (between January 2005 and June 2007) (15). The only exclusion criteria was living in a residential aged care facility. Dietary data were first collected in the third wave of CHAMP (between August 2010 and August 2013; baseline in the present study) with 794 men aged 75 years and over (16). Of these men, 782 (98.4%) had MACE and medical history data. A total of 243 men had a history of myocardial infarction (MI), stroke, CCF, and/or coronary revascularisation identified from self-reported questionnaires at third wave and data linkage of MACE from first wave up to third wave of CHAMP. Thus, a total of 539 men were included in the prospective analyses on associations between dietary iron intakes and MACE. Of these, 523 men had haemoglobin and 522 men had serum iron data available for cross-sectional analyses on the associations between dietary iron intakes and iron status biomarkers. Flowchart of participants' inclusion is shown in Figure 1. The CHAMP study was approved by the Concord Hospital Human Research Ethics Committee (HREC/14/CRGH/17), and participants provided written informed consent for all assessments.

### Dietary Intake

Dietary data was collected using a validated dietitian-administered diet history questionnaire, involving questions about usual dietary intake in the previous 3 months and used food models, photographs, and household measures to estimate amounts consumed (16, 17). A food checklist was included, and relatives, carers, and/or family members of participants were encouraged to attend the interview to aid memory recall (16). The validity of this diet history questionnaire by comparison with a prospective 4-day weighed food record which does not rely on participants' memory was previously reported in a subgroup of 56 CHAMP men (16). The mean differences in dietary intakes between the two methods were generally less than 20%, and specifically for iron intake was 1% (16). Entries were converted to foods, food groups, and total iron intakes using FoodWorks 7 Professional for Windows (Xyris Software (Brisbane, Australia) Pty Ltd) and The Australian Food, Supplement and Nutrient Database 2007 (AUSNUT 2007) (18). Food groups included fruits, vegetables, grains, dairy/alternatives, and meat/alternatives. Food subgroups of HI sources included red meat, poultry, processed meat, seafood, and offal.

HI and NHI intakes are not available in AUSNUT 2007 (18). Thus, the dietary calculation was used to derive HI and NHI intakes from total iron intakes with reference to previous literature published by Rangan et al. (19) the most recent Australian data available. The average proportions of HI content in the Australian food supply (19), for different food sources were used: pork (65%), poultry (62%), beef and lamb (61%), seafood (40%) and offal (33%). HI intake from a single food source, was calculated as the amount of the food source consumed multiplied by the proportion of HI content in the food source. HI intake from a single food source in a recipe, was calculated as the amount of the food source consumed multiplied by the proportion of the single food source contributing to the total iron of the recipe (excluding ingredient sources not containing any iron such as canola oil and white sugar) multiplied by the proportion of HI content in the food source. HI intake from a recipe was calculated as the sum of the HI from all food sources containing HI in the recipe. NHI intake from a single food source or recipe was calculated as the total iron intake minus HI intake. Iron in all other food sources and recipes not containing HI, were assumed to contain 100% of total iron from NHI.

### MACE Measurement

MACE outcomes are a composite index. Five-point MACE is comprised of all-cause mortality, MI, CCF, coronary revascularisation, and/or ischaemic stroke. Four-point MACE includes the four endpoints of MI, CCF, coronary revascularisation, and/or ischaemic stroke, and excludes all-cause mortality. Individual endpoints of MACE were censored on 31 December 2017 (7.4 years following nutrition assessment).

MACE data were obtained through data linkage from three population databases: New South Wales Admitted Patient Data Collection Registry; Births, Deaths and Marriages Registry; and Australian Bureau of Statistics Mortality Data using International Classification of Diseases coding (ICD-10) for the following: MI (codes: I21.0–I21.4, I21.9, I22.0–I22.1, I22.9); ischaemic stroke (codes: I63.0–I63.6, I63.8–I63.9); CCF (codes: I50.0–I50.1, I50.9). The Australian Classification of Health Interventions codes were used to identify participants who underwent coronary revascularisation, including coronary artery angioplasty (codes: 38300-00, 38300-01, 38303-00, 38303-01, 35309-06, 35309-07, 90218-00 to 90218-03), or coronary artery bypass grafting (codes: 38497-00 to 38497-07, 38500-00 to 38500-03, 38503-00, 38503-01, 38503-04, 38503-05, 90201-00 to 90201-03). Data on cause-specific mortality was not available.

### Measurements of haemoglobin and serum iron

Fasting blood samples were collected from participants on the morning of their clinic visit at third wave. Blood tests were performed at the Diagnostic Pathology Unit of Concord Hospital, which is a National Australian Testing Authority-accredited pathology service, using a Modular Analytics system (Roche Diagnostics, Castle Hill, Australia). Haemoglobin and serum iron were measured by absorption spectrophotometry.

### Other Measurements

Data on anthropometry, socio-demographics, lifestyle, and health factors were collected through self-reported questionnaire, interviewer-administered questionnaire, biochemical analyses, medication inventory, and physical measurements at third wave. Height, weight, waist circumference, and hip circumference were measured following standardised protocols as previously described (20). BMI was calculated as kg/m^2^. Physical activity was assessed through Physical Activity Scale for the Elderly (PASE) (21).

Marital status was categorised into ‘married/de facto' and ‘not married/divorced/separated/widowed/never married/other'. ‘Age Pension only' referred to those who only received the Age Pension, whilst ‘other' referred to those with other sources of income apart from the Age Pension including veteran pension, repatriation pension, superannuation, private income, business ownership, farm ownership, business partnership, wage, salary, and/or other. Country of birth was categorised into ‘Australia', ‘Greece/Italy', and ‘other'. Smoking status was categorised into ‘non-smoker', ‘ex-smoker', and ‘current smoker' based on self-reported smoking history. Alcohol consumption was categorised as ‘non-drinker' for those who had <12 standard (std) drinks in their entire life, ‘ex-drinker' for those had <12 std drinks in the past 12 months, ‘safe drinker' for those who had ≤4 std drinks per day and ≤10 std drinks per week, and ‘harmful drinker' for those who had >4 std drinks per day or >10 std drinks per week (22). The Mediterranean diet score was generated based on previous literature (23) and indicated the overall dietary pattern in the present study. The Mediterranean diet score was comprised of 9 food category components reflecting adherence to the Mediterranean dietary pattern and each category scored out of 2 with an overall possible maximum score of 18, where 0 is considered lowest adherence and 18 is considered highest adherence (23).

Prescription and nonprescription medication used daily or almost daily were brought to the clinic visit and recorded. Participants were asked whether they had taken any other medications during the past month. Reported medications on the interviewer-administered questionnaire were used to determine the number of medications and were coded using the Iowa Drug Information Service drug code numbers. Iron and/or multivitamin supplement use was categorised into ‘yes' and ‘no'. Use of relevant medications was categorised into ‘non-steroidal anti-inflammatory drug (NSAID), anticoagulant and/or antiplatelet only', ‘proton pump inhibitor (PPI) and/or histamine-2 receptor antagonist (H2RA) only', ‘NSAID, anticoagulant and/or antiplatelet with PPI and/or H2RA', and ‘neither NSAID, antiplatelet, anticoagulant, PPI or H2RA'. Self-rated health was obtained through response to the question, ‘compared to other people of your age, how would you rate your health?', and data was categorised into ‘very poor/poor/fair' and ‘good/excellent'. Cognitive function was assessed using the Mini-Mental State Examination (MMSE) (24), which has a score range from 0 to 30 (higher scores indicating better cognition). Participants with a score of less than 24 were categorised as ‘cognitively impaired', which has a sensitivity of 0.85 and specificity of 0.90 in studies of older adults in community settings (25). Anaemia was defined as haemoglobin levels <130g/L (26). Low serum iron was defined as ≤13µmol/L based on a study that used bone marrow iron depletion to diagnose iron deficiency (27). The inflammatory biomarker included was cytokine interleukin-6 (IL-6). Serum creatinine (Scr) levels in µmol/L multiplied by 0.0113 to convert to mg/dL, were used to estimate glomerular filtration rate (eGFR). We used the Chronic Kidney Disease Epidemiology Collaboration (CKD-EPI) equations for men (28) as follows: Scr (µmol/L/mg/dL) = ≤80/≤ 0.9: eGFR = 141 × (Scr/0.9)-0.411 × (0.993) Age and Scr > 80/> 0.9, eGFR = 141 × (Scr/0.9)-1.209 × (0.993)Age. Chronic kidney disease was defined as eGFR <60mL/min/1.73m2. The number of comorbidities was determined by the sum of all self-reported conditions including: diabetes, thyroid disease, osteoporosis, Paget's disease, stroke, Parkinson's disease, kidney stones, dementia, depression, epilepsy, hypertension, angina, intermittent claudication, chronic obstructive pulmonary disease, liver disease, renal disease, arthritis, gout, and cancer (excluding nonmelanoma skin cancers).

Participants were classified as robust if they had none, pre-frail if they had one or two, and frail if they had three or more of the five frailty components. Frailty was defined using the Fried frailty phenotype criteria according to the Cardiovascular Health Study (CHS) for weakness and slowness (29), and adapted criteria for weight loss, exhaustion, and low activity previously described (30).

### Statistical Analysis

Statistical analysis was carried out using SPSS software version 25 (IBM Corp., Armonk, NY, USA) (31). Normality tests (histogram, Q-Q plot and Shapiro-Wilk test) conducted found that most data had a skewed distribution. Descriptive characteristics were expressed as median (interquartile range) (IQR) and as number of participants (percentage of participants). Participant characteristics according to dietary HI as a percentage of total iron intake were compared. For categorical data, chi-square tests were used to compare participant characteristics across tertiles. For numerical data, median tests, and Bonferroni correction for multiple tests were used to compare participant characteristics between each pair of tertiles.

The associations between iron intakes as continuous variables with continuous serum iron and haemoglobin, were evaluated through linear regression. Results are presented as ß coefficients with 95% confidence intervals (CIs) for linear regression. Models were adjusted by covariates, including socio-demographic and lifestyle factors (age, BMI, country of birth, marital status, age pension, alcohol consumption, smoking status, PASE, energy intake, Mediterranean diet score, number of serves of fruits, vegetables, grains, meat/alternatives, dairy/alternatives, and iron and/or multivitamin supplement use, and health (number of medications, frailty status, number of comorbidities, and IL-6). For serum iron, the fully adjusted model was conducted with and without haemoglobin. Collinearity diagnostics was conducted and there was no collinearity as variance inflation factors (VIFs) for independent variables were <2.5 in all analyses (32).

The Cox regression method was used to determine the association between iron intakes with five-point MACE, four-point MACE excluding all-cause mortality, and individual endpoints. Results are presented as hazard ratios (HRs) with 95% CI. Iron intakes were analysed as both continuous and categorical variables (i.e. categorised into tertiles with the lowest as the reference category). The proportional hazards assumption was determined using the graphical method (‘log minus log'). Time interaction with each included variable was tested and none were significant. Pre-specified potential confounders considered for inclusion in the Cox regression models were socio-demographic and lifestyle factors (age, BMI, country of birth, marital status, age pension, alcohol consumption, smoking status, PASE, energy intake, Mediterranean diet score, number of serves of fruits, vegetables, grains, meat/alternatives, dairy/alternatives, and iron and/or multivitamin supplement use), and health (NSAID, anticoagulant, antiplatelet, PPI and/or H2RA use, frailty status, diabetes, CKD, cancer, IL-6, and haemoglobin). Using backward elimination with likelihood ratio tests on dietary iron intakes with each outcome, the covariates included in the multivariable model were: age, BMI, country of birth, age pension, energy intake, number of serves of vegetables, fruit, meat/alternatives, grains, NSAID, anticoagulant, antiplatelet and/or PPI or H2RA use, haemoglobin, frailty status, and CKD. Interactions between covariates in the finally adjusted model were tested and none were significant. For all analyses, any p values < 0.05 or 95% CIs not including the null point were considered statistically significant. All covariates were included in adjusted models assessing associations between iron intakes with five-point MACE, four-point MACE excluding all-cause mortality, all-cause mortality, CCF, and ischaemic stroke. NSAID, anticoagulant, antiplatelet, PPI and/or H2RA for MI, and frailty status for coronary revascularisation could not be included in adjusted models assessing dietary iron intakes and those outcomes due to low numbers experiencing an event.

Survival analysis plots of event-free survival stratified by tertiles of iron intakes for unadjusted and adjusted analyses were generated. For dietary iron intakes as continuous variables associated with five-point MACE and/or four-point MACE excluding all-cause mortality in fully adjusted analyses, the scale of the relevant continuous dietary iron intake was estimated through plotting midpoints of deciles of the dietary iron intake versus beta coefficients for each decile derived from Cox regression (33). Midpoints of deciles were used instead of midpoints of quartiles for greater sensitivity with more data points (33). Curve estimation analyses was then conducted with the R-squared value indicating variance in the dependent variable explained by the regression model, where 0 represents 0% of variance explained and 1 represents 100% of variance explained.

### Sensitivity analysis on food subgroup intakes

Sensitivity analyses were conducted to additionally adjust for dietary factors from food subgroups containing HI that may confound the associations between dietary iron intakes and MACE, including the number of serves of red meat, poultry, processed meat, and seafood. Offal was consumed minimally and was not included as a covariate.

## Results

A total of 539 men without a history of MI, stroke, CCF, and/or coronary revascularisation had iron intake and MACE data available at nutrition assessment. The median participant age was 80.0 (IQR 77.0–83.0) years and BMI was 27.3 (IQR 25.0–30.1) kg/m^2^. A substantial proportion of participants were anaemic (14.5%) or had low serum iron (22.8%). The median intakes of dietary iron intakes were: 12.7 (IQR 10.4–16.0) mg for total iron, 10.9 (IQR 8.7–13.8) mg for NHI, and 1.7 (IQR 1.2–2.4) mg for HI. Table 1 presents the participant characteristics and Table 2 presents dietary intake according to tertiles of HI as a percentage of total iron intake. Most characteristics did not differ between the tertiles. By definition, HI and NHI intakes were different between the HI as a percentage of total iron intake tertiles. The top tertile of HI as a percentage of total iron intake was less likely to have been non-drinkers and ex-drinkers, and more likely to have been safe and harmful drinkers. Participants in the top tertile of HI as a percentage of total iron intakes also had lower energy intake than the middle tertile, and a lower Mediterranean diet score, lower intakes of vegetables and grains, higher intakes of poultry and processed meat than the bottom tertile. Those in the top tertile of HI as a percentage of total iron intakes, also had higher BMI, lower total iron intake, lower intake of dairy/alternatives, and higher intake of meat/alternatives and red meat than those in both the bottom and middle tertiles.

**Table 1:** Participant characteristics (median and interquartile range; percentages and number of participants) according to haem iron as a percentage of total iron intake tertiles (n = 539)

**Variables**	**All n = 539**	**Bottom tertile ≤10.67%; n = 180**	**Middle tertile 10.68–16.14%; n = 180**	**Top tertile ≥16.15%; n = 179**	**P value**^**1**^
Age (years)	80.0 (77.0–83.0)	80.0 (77.0–83.0)	80 (77.0–83.8)	80.0 (77.0–83.0)	.89
BMI (kg/m2) (n = 533)	27.3 (25.0–30.1)	26.5 (24.7–29.4)^a^,^b^	27.5 (25.3–30.4)^a^	27.8 (24.9–30.5)^b^	.020^a^ .018^b^
Waist circumference (cm) (n = 535)	101.3 (94.2–107.8)	100.5 (95.8–105.7)	102.9 (98.7–109.3)	102.0 (98.3–108.0)	.14
Hip circumference (cm) (n = 535)	101.9 (97.5–107.6)	100.5 (95.9–105.7)	102.9 (98.7–109.3)	102.0 (98.3–108.0)	.11
Marital status (n = 536)					.58
Married	418 (78.0)	135 (76.3)	145 (80.6)	138 (77.1)	
Not married	118 (22.0)	42 (23.7)	35 (19.4)	41 (22.9)	
Source of income (n = 538)					.73
Age Pension only	213 (39.6)	70 (38.9)	68 (38.0)	75 (41.9)	
Other	325 (60.4)	110 (61.1)	111 (62.0)	104 (58.1)	
Countiy of birth					.063
Australia	279 (51.8)	84 (46.7)	109 (60.6)	86 (48.0)	
Greece/Italy	131 (24.3)	46 (25.6)	36 (20.0)	49 (27.4)	
Other	129 (23.9)	50 (27.8)	35 (19.4)	44 (24.6)	
Cigarette smoking status (n = 536)					.52
Non-smoker	231 (43.1)	78 (43.3)	82 (46.1)	71 (39.9)	
Ex-smoker	286 (53.4)	98 (54.4)	90 (50.6)	98 (55.1)	
Current smoker	19 (3.5)	4 (2.2)	6 (3.4)	9 (5.1)	
Alcohol consumption (n = 538)					.009
Non-drinker	52 (9.7)	22 (12.2)	18 (10.0)	12 (6.7)	
Ex drinker	67 (12.5)	32 (17.8)	22 (12.2)	13 (7.3)	
Safe drinker	225 (41.8)	76 (42.2)	68 (37.8)	81 (45.5)	
Harmful drinker	194 (36.1)	50 (27.8)	72 (40.0)	72 (40.4)	
Iron and/or multivitamin supplement use	10 (1.9)	4 (2.2)	1 (0.6)	5 (2.8)	.26
Medication use					.29
NSAID, anticoagulant and/or antiplatelet only	177 (32.8)	62 (34.4)	58 (32.2)	57 (31.8)	
PPI and/or H2RA only	75 (13.9)	22 (12.2)	22 (12.2)	31 (17.3)	
NSAID, anticoagulant and/or antiplatelet with PPI and/or H2RA	66 (12.2)	29 (16.1)	21 (11.7)	16 (8.9)	
Neither NSAID, anticoagulant, antiplatelet, PPI and/or H2RA	221 (41.0)	67 (37.2)	79 (43.9)	75 (41.9)	
Number of medications	4.0 (2.0–6.0)	4.0 (2.0–6.0)	3.0 (2.0–5.0)	3.0 (2.0–5.0)	.19
Anaemia (n = 523)	76 (14.5)	32 (18.4)	22 (12.6)	22 (12.6)	.21
Haemoglobin (g/L) (n = 523)	144.0 (136.0–152.0)	143.0 (132.8–150.0)	145.5 (136.0–153.3)	146.0 (136.0–155.0)	.031t
Serum iron (umol/L) (n = 522)	17.0 (14.0–21.0)	17.0 (14.0–20.0)	18.0 (13.0–22.0)	17.0 (14.0–20.3)	.39
Low serum iron (n = 522)	119 (22.8)	36 (20.7)	45 (25.9)	38 (21.8)	.48
Interleukin-6 (pg/mL) (n = 492)	2.5 (1.2–5.0)	2.4 (1.2–4.3)	2.3 (1.3–4.8)	3.0 (1.3–5.6)	.22
MMSE (n = 511)	28.0 (26.0–29.0)	28.0 (26.0–30.0)	28.0 (27.0–29.0)	28.0 (26.0–29.0)	.99
Cognitively impaired (n = 511)	45 (8.8)	15 (8.8)	10 (5.7)	20 (12.0)	.12
Self-rated health					.77
Very poor/poor/fair	113 (21.0)	41 (22.8)	36 (20.0)	36 (20.1)	
Good/excellent	426 (79.0)	139 (77.2)	144 (80.0)	143 (79.9)	
Number of comorbidities	2.0 (1.0–3.0)	2.0 (1.0–3.0)	2.0 (1.0–3.0)	2.0 (1.0–3.0)	.52
Diabetes Mellitus	105 (19.5)	43 (23.9)	32 (17.8)	30 (16.8)	.18
Chronic Kidney Disease (n = 525)	171 (32.6)	58 (33.1)	55 (31.4)	58 (33.7)	.93
Cancer	42 (7.8)	11 (6.1)	17 (9.4)	14 (7.8)	.50
Frailty Status (n = 534)					.97
Robust	266 (49.8)	91 (51.1)	89 (50.3)	86 (48.0)	
Pre-frail	246 (46.1)	79 (44.4)	81 (45.8)	86 (48.0)	
Frail	22 (4.1)	8 (4.5)	7 (4.0)	7 (3.9)	
PASE	131.7 (88.5–166.7)	135.9 (88.7–163.6)	130.7 (82.7–166.1)	131.7 (94.6–171.7)	.63

Notes: IQR = interquartile range; BMI = body mass index; Std = standard; NSAID = non-steroidal anti-inflammatory drug; PPI = proton pump inhibitor; H2RA = histamine-2 receptor antagonist; MMSE = Mini-Mental State Examination; eGFR = estimated glomerular filtration rate; PASE = Physical Activity Scale for the Elderly. ^1^P values were obtained using the median test and Bonferroni correction for multiple tests to compare all haem iron as a percentage of total iron intake tertile groups for differences in median values of continuous variables. Differences between pairs of tertile groups are denoted by each letter a, b, or c. ^1^P values were obtained using the chi-square test to compare all haem iron as a percentage of total iron intake tertile groups for differences in proportions of participants in categories for categorical variables. tNo differences between groups were observed after Bonferroni correction for multiple test

**Table 2:** Dietary intake (median and interquartile range) according to haem iron as a percentage of total iron intake tertiles (n = 539)

**Variables**	**All n = 539**	**Bottom tertile ≤10.67%; n = 180**	**Middle tertile 10.68–16.14%; n = 180**	**Top tertile ≤16.15%; n = 179**	**P value**^**1**^
Energy intake (kJ)	8862.9 (7312.2–10549.7)	8982.9 (7334.2–10557.9)	9210.1 (7778.2–10557.9)^a^	8448.2 (6957.3–10004.6)a	.007^a^
Mediterranean diet score	8.0 (6.8–9.4)	8.7 (7.4–10.0)^a^,^b^	7.9 (6.8–9.1)^a^	7.6 (6.2–9.1)^b^	.002^a^ .001^b^
Fruits (serves/d)	1.8 (1.1–2.7)	2.0 (1.2–3.1)	1.9 (1.1–2.7)	1.6 (0.9–2.3)	.072
Vegetables (serves/d)	3.5 (2.5–4.9)	3.7 (2.5–5.3)^a^	3.6 (2.6–4.7)	3.1 (2.3–4.6)^a^	.029^a^
Grains (serves/d)	5.0 (3.8–6.5)	5.5 (4.3–7.2)^a^,^b^	4.9 (3.7–6.3)^a^	4.4 (3.2–5.8)^b^	.018^a^ .001^b^
Meat/alternative (serves/d)	2.9 (2.2–3.7)	2.4 (1.7–3.1)^a^,^b^	2.8 (2.3–3.8)^a^,^c^	3.3 (2.6–4.0)^b^,^c^	.001 ^a^ p <.001^b^ .003^c^
Dairy/alternative (serves/d)	1.7 (1.0–2.4)	1.8 (1.2–2.6)^a^	1.9 (1.3–2.6)^b^	1.4 (0.9–2.2)^a^,^b^	.002^a^ <.001^b^
Red meat (serves/d)	1.1 (0.7–1.6)	0.7 (0.4–0.9)^a^,^b^	1.2 (0.9–1.5) ^a^,^c^	1.7 (1.2–2.2) ^b^,^c^	<.001^a^,^b^,^c^
Poultry (serves/d)	0.3 (0.2–0.6)	0.3 (0.1–0.5)^a^,^b^	0.3 (0.2–0.6)^a^	0.4 (0.2–0.6)^b^	.018^a^ .001^b^
Processed meat (serves/d)	0.1 (0.0–0.2)	0.1 (0.0–0.2)^a^,^b^	0.1 (0.0–0.3)^a^	0.1 (0.0–0.3)^b^	.009^a^ .011^b^
Seafood (serves/d)	0.3 (0.2–0.5)	0.3 (0.1–0.5)	0.3 (0.2–0.5)	0.3 (0.2–0.5)	.66
Total iron (mg/d)	12.7 (10.4–16.0)	14.1 (11.0–18.2)^a^	12.9 (10.9–15.6)^b^	11.6 (9.7–14.1)^a^,^b^	<.001^a^ .008^b^
Haem iron (mg/d)	1.7 (1.2–2.4)	1.1 (0.8–1.4)^a^,^b^	1.7 (1.4–2.2)^a^,^c^	2.5 (2.0–3.1)^b^,^c^	<.001^a^,^b^,^c^
Non-haem iron (mg/d)	10.9 (8.7–13.8)	12.9 (10.1–16.1)^a^,^b^	11.2 (9.4–13.6)^a^,^c^	9.0 (7.4–11.1)^b^,^c^	.002^a^ <.001^b^,^c^

Notes: IQR = interquartile range. ^1^P values were obtained using the median test and Bonferroni correction for multiple tests to compare all haem iron as a percentage of total iron intake tertile groups for differences in median values of continuous variables. Differences between pairs of tertile groups are denoted by each letter a, b, or c. †No differences between groups were observed after Bonferroni correction for multiple tests

### Cross-sectional analyses on haemoglobin and serum iron

Dietary iron intake as continuous variables were not associated with serum iron and haemoglobin in all linear regression analyses (see Supplementary Tables 1 and 2 respectively).

### Longitudinal analyses on MACE

**Figure 2:**
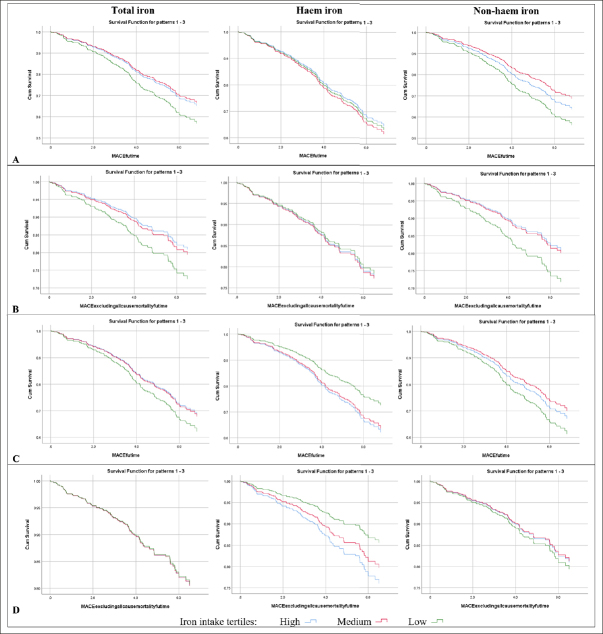
Survival free of MACE: unadjusted analyses (A) Five-point MACE and (B) Four-point MACE excluding all-cause mortality; and fully adjusted analyses (C) Five-point MACE and (D) Four-point MACE excluding all-cause mortality MACE = major adverse cardiovascular event; Cum = cumulative; Futime = follow-up time.

During the median follow-up of 5.3 (IQR 4.6–6.3) years, the incidences were: five-point MACE 31.2% (n = 168); four-point MACE excluding all-cause mortality 17.8% (n = 96); all-cause mortality 20.1% (n = 111); CCF 11.3% (n=61); MI 3.7% (n = 20); ischaemic stroke 3.2% (n = 17); and coronary revascularisation 3.1% (n = 15). The number of events for MACE and individual endpoints according to tertiles of dietary iron intakes are available in Supplementary Table 3. Univariate analyses of predictors of MACE are shown in Supplementary Table 4. Survival analysis plots on MACE stratified by tertiles of dietary pattern scores are shown in Figure 2 for unadjusted and fully adjusted analyses. The associations between dietary iron intakes and MACE are presented in Table 3.

**Table 3:** Associations between dietary iron intakes and major adverse cardiovascular events (MACE) and individual endpoints of MACE using Cox regression presented as hazard ratios (95% CI) (n = 539)

**Iron intake**	**Bottom tertile (reference category)**	**Middle tertile**	**Top tertile**	**As continuous variable**
**Total iron**^**a**^	**≤11.26mg/d**	**11.27–14.75mg/d**	**≥14.76mg/d**	**+1mg/d**
Five-point MACE				
Model 1	1	0.73 (0.50, 1.05) P = .088	0.76 (0.53, 1.09) P = .14	1.01 (0.99, 1.02) P = .32
Model 2	1	0.81 (0.54, 1.21) P = .30	0.83 (0.52, 1.33) P = .44	1.01 (0.99, 1.02) P = .50
Model 3	1	0.82 (0.54, 1.24) P = .35	0.80 (0.50, 1.29) P = .37	1.00 (0.99, 1.02) P = .59
Four-point MACE excluding all-cause mortality				
Model 1	1	0.72 (0.44, 1.16) P = .17	0.66 (0.41, 1.08) P = .098	0.99 (0.97, 1.02) P = .62
Model 2	1	0.98 (0.57, 1.67) P = .94	1.07 (0.57, 2.00) P = .84	1.00 (0.98, 1.03) P = .81
Model 3	1	1.01 (0.59, 1.75) P = .96	1.02 (0.54, 1.92) P = .97	1.00 (0.98, 1.03) P = .94
**Haem iron**^**b**^	**≤1.40mg/d**	**1.41–2.10mg/d**	**≥2.11mg/d**	**+1mg/d**
Five-point MACE				
Model 1	1	1.06 (0.73, 1.52) P = .78	0.95 (0.66, 1.39) P = .80	1.04 (0.88, 1.22) P = .66
Model 2	1	1.31 (0.87, 1.98) P = .20	1.44 (0.90, 2.30) P = .13	1.45 (1.16, 1.81) P = .001
Model 3	1	1.41 (0.92, 2.17) P = .11	1.49 (0.92, 2.40) P = .11	1.45 (1.16, 1.80) P = .001
Four-point MACE excluding all-cause mortality				
Model 1	1	1.07 (0.65, 1.74) P = .80	1.05 (0.64, 1.71) P = .86	1.11 (0.90, 1.37) P = .32
Model 2	1	1.43 (0.83, 2.47) P = .20	1.74 (0.95, 3.19) P = .075	1.65 (1.26, 2.18) P <.001
Model 3	1	1.46 (0.83, 2.58) P = .19	1.76 (0.95, 3.29) P = .075	1.64 (1.26, 2.15) P <.001
**Non-haem iron**^**c**^	**≤9.42mg/d**	**9.43–12.77mg/d**	**≥12.78mg/d**	**+1mg/d**
Five-point MACE				
Model 1	1	0.65 (0.45, 0.95) P = .027	0.79 (0.55, 1.12) P = .19	0.98 (0.95, 1.02) P = .38
Model 2	1	0.71 (0.47, 1.08) P = .11	0.83 (0.52, 1.33) P = .44	0.99 (0.95, 1.04) P = .67
Model 3	1	0.73 (0.48, 1.11) P = .14	0.82 (0.51, 1.32) P = .41	0.98 (0.93, 1.02) P = .32
Four-point MACE excluding all-cause mortality				
Model 1	1	0.67 (0.41, 1.08) P = .10	0.64 (0.39, 1.04) P = .072	0.97 (0.92, 1.02) P = .29
Model 2	1	0.88 (0.52, 1.50) P = .63	0.94 (0.50, 1.78) P = .86	1.02 (0.96, 1.09) P = .47
Model 3	1	0.90 (0.52, 1.55) P = .70	0.91 (0.48, 1.75) P = .79	1.01 (0.95, 1.07) P = .80

Notes: Model 1 unadjusted (n = 539 for total, 168 five-point MACE, and 96 four-point MACE excluding all-cause mortality); Model 2 adjusted by age (continuous), BMI (continuous), country of birth (Australia v. Greece/Italy v. other), age pension (only v. other), energy intake (continuous), number of serves of vegetables (continuous), fruit (continuous), meat/alternatives (continuous), grains (continuous) (n = 532 for total, 164 five-point MACE, and 95 four-point MACE excluding all-cause mortality); Model 3 adjusted by Model 2 plus NSAID, anticoagulant, antiplatelet and/or PPI or H2RA use (NSAID, anticoagulant and/or antiplatelet only v. PPI and/or H2RA only v. NSAID, anticoagulant and/or antiplatelet with PPI and/or H2RA v. neither NSAID, antiplatelet, anticoagulant, PPI or H2RA), haemoglobin (continuous), frailty status (robust v. pre-frail v. frail), and CKD (yes v. no) (n = 516 for total, 160 five-point MACE, and 92 four-point MACE excluding all-cause mortality). a. Bottom tertile ≤11.26mg/d, n = 180 with median (IQR) 9.59 (8.24, 10.41); middle tertile 11.27–14.75mg/d, n = 180 with median (IQR) 12.71 (11.92, 13.66); top tertile ≥14.76mg/d, n = 179 with median (IQR) 17.64 (15.98, 20.13). b. Bottom tertile ≤1.40mg/d, n = 180 with median (IQR) 1.00 (0.75, 1.21); middle tertile 1.41–2.10mg/d, n = 180 with median (IQR) 1.74 (1.58, 1.92); top tertile ≥2.11mg/d, n = 179 with median (IQR) 2.65 (2.38, 3.14). c. Bottom tertile ≤9.42mg/d, n = 180 with median (IQR) 7.93 (6.72, 8.75); middle tertile 9.43–12.77mg/d, n = 180 with median (IQR) 10.86 (10.15, 11.62); top tertile ≥12.78mg/d, n = 179, with median (IQR) 15.30 (13.81, 17.55)

### Total iron

Total iron intake was not associated with five-point MACE and four-point MACE excluding all-cause mortality in unadjusted and adjusted analyses (Table 3).

### Haem iron

HI intake as a continuous variable (per 1mg increment) was not associated with five-point MACE and four-point MACE excluding all-cause mortality in unadjusted analyses but were both associated with increased risk in fully adjusted analysis (HR 1.45 [95% CI: 1.16, 1.80, P = .001] and HR 1.64 [95% CI: 1.26, 2.15, P <.001] respectively). Supplementary Figure 1 shows the linear relationship between HI intake and MACE through plotting decile midpoints of HI intake versus decile beta coefficients from fully adjusted analyses. HI intake as categorical variables were not associated with five-point MACE and four-point MACE excluding all-cause mortality in unadjusted and adjusted analyses.

### Non-haem iron

Compared with the bottom tertile of NHI intake (≤9.42mg), the middle tertile (9.43–12.77mg) was associated with reduced risk of five-point MACE in unadjusted analysis (HR 0.65 [95% CI: 0.45, 0.95, P = .027]) but was not associated in fully adjusted analysis. Higher NHI intake (per 1mg increment) was not associated with five-point MACE in unadjusted and adjusted analyses. NHI intake as both categorical and continuous variables was not associated with four-point MACE excluding all-cause mortality in unadjusted and adjusted analyses.

### Longitudinal analyses on individual endpoints of MACE

Survival analysis plots on individual endpoints of MACE stratified by tertiles of iron intakes are shown in Supplementary Figure 2 for unadjusted analyses and Supplementary Figure 3 for fully adjusted analyses. The associations between dietary iron intakes and individual endpoints of MACE are presented in Supplementary Table 5.

Total iron intakes were not associated with individual endpoints of MACE in unadjusted and adjusted analyses.

Higher HI intake (per 1 mg increment) was not associated with all-cause mortality and CCF in unadjusted analyses but was associated with increased risk in the fully adjusted analyses (HR: 1.51 [95% CI: 1.15, 1.99, P = .003] and HR 2.08 [95% CI: 1.45, 2.98, P <.001] respectively). Higher HI intake (per 1mg increment) was associated with increased coronary revascularisation in unadjusted analysis (HR 1.88 [95% CI: 1.30, 2.72, P = .001]), which remained in the fully adjusted model (HR: 1.89 [95% CI: 1.15, 3.10, P = .012]). Compared with the bottom tertile (≤1.40mg/d), both the middle (1.41–2.10mg/d) and top tertiles (≥2.11mg/d) of HI intake were not associated with CCF in unadjusted analyses, however, were associated with increased risks in fully adjusted analyses (HR: 3.10 [95% CI: 1.43, 6.73, P = .004] and HR: 3.07 [95% CI: 1.28, 7.35, P = .012] respectively). HI intake as categorical variables were not associated with all-cause mortality, coronary revascularisation, MI, and ischaemic stroke in unadjusted and adjusted analyses.

Compared with the bottom tertile of NHI intake (≤9.42mg), the middle tertile of NHI intake (9.43–12.77mg) was associated with reduced risk of all-cause mortality in unadjusted analysis (HR 0.60 [95% CI: 0.38, 0.97, P = .037]), which remained in the fully adjusted model (HR: 0.56 [95% CI: 0.33, 0.96, P = .035]). Higher NHI intake (per 1mg increment) was not associated with all-cause mortality in unadjusted and adjusted analyses. NHI intake as continuous and categorical variables were not associated with CCF, coronary revascularisation, MI, and ischaemic stroke in unadjusted and adjusted analyses.

### Sensitivity analysis on food subgroup intakes

After additional adjustment for food subgroup intakes (red meat, poultry, processed meat, and seafood) higher HI intake (per 1mg increment) remained associated with increased risks of five-point MACE, four-point MACE excluding all-cause mortality, all-cause mortality, CCF, and coronary revascularisation (see Supplementary Table 6). Compared with the bottom tertile, the middle tertile of HI intake also remained associated with increased risk of CCF. The middle tertile of NHI intake remained associated with reduced risk of all-cause mortality.

## Discussion

To our knowledge, this is the first prospective cohort study to evaluate the associations between dietary iron intakes (total iron, HI, and NHI) with MACE and individual endpoints of MACE in older men aged 75 years and over. In longitudinal analyses over 5 years, we found that consuming higher HI intakes were associated with increased five-point MACE, four-point MACE excluding all-cause mortality, all-cause mortality, CCF, and coronary revascularisation which remained after additional adjustment for food subgroup intakes of red meat, processed meat, poultry, and seafood. In contrast, the middle tertile of NHI intake remained associated with reduced risk of all-cause mortality. Total iron intakes were not associated with MACE and individual endpoints. Dietary iron intakes were not cross-sectionally associated with iron status biomarkers.

In agreement with most previous study findings, dietary iron intakes were not associated with serum iron and haemoglobin. In another cross-sectional analysis, dietary total iron intakes were not associated with serum iron, but was negatively associated with haemoglobin in older adults aged 65 years and over (34). Dietary total iron intakes were also not cross-sectionally associated with iron status parameters in older adults aged 65 years and over (including haemoglobin and plasma iron) (14), in older adults aged 70 years and over (including haemoglobin and serum iron) (12), and in older adults aged 80 years and over (including serum iron) (35). The inconsistencies and lack of association between dietary iron intakes and iron status biomarkers, could be explained by interindividual variation in the bioavailability of iron due to different meal compositions with potential enhancers and inhibitors present at each meal that are difficult to account for (13, 36), and increased absorption efficiency in the state of iron deficiency (37). Furthermore, for serum iron, diurnal variation is varied between individuals (38) and longer periods of fasting prior to specimen collection have been associated with elevated serum iron levels (39).

Higher HI intakes were associated with increased five-point MACE, four-point MACE excluding all-cause mortality, all-cause mortality, CCF, and coronary revascularisation, whilst total iron and NHI intakes were not associated with MACE and individual endpoints, except the middle tertile of NHI intake when compared with the bottom tertile was associated with reduced risk of all-cause mortality. This agrees with previous research which found no associations between dietary total iron intakes with CHD mortality over 14 years in adults aged 45–64 years (40), and with CHD and acute myocardial infarction over 13 years in adults aged 40–74 years (41). Similarly, in men aged 40–75 years, dietary total iron and NHI intakes were not associated with risks of CHD, however, compared with the lowest quintile of HI intake, the highest quintile was associated with higher risk of fatal coronary disease over 4 years (42). In adults aged 50–71 years, compared with the lowest quintile of HI intake, the highest quintile was independently associated with increased risks of all-cause mortality and cardiovascular mortality over 16 years (43). In adults aged 45–84 years, NHI intakes was not associated with CVD over 6 years, but compared with the lowest quintile, the highest quintile of HI intake was associated with increased risk (44). In a meta-analysis of prospective cohort studies involving adults aged 18 years and over with a follow-up range of 4 to 15 years, total iron and NHI intakes were not associated with CVD, but HI intake was associated with increased risk when the lowest category was compared to the highest category and per 1mg increment (10). In another meta-analysis of prospective cohort studies in adults aged 18 years and over with a follow-up range of 4 to 16 years, total iron and NHI intakes were not associated with cardiovascular mortality (death due to CVD, stroke, CHD, and myocardial infarction), whilst HI intake was associated with increased risk when the lowest category was compared to the highest category and per 1mg increment (11).

Similar to the present study where higher HI intakes remained associated with increased five-point MACE, four-point MACE excluding all-cause mortality, all-cause mortality, CCF, and coronary revascularisation after additional adjustment for food subgroup intakes including red meat, a study found that even after adjusting for red meat intake HI intake was associated with increased type 2 diabetes mellitus, a risk factor of CVD over 11 years in adults aged 45–74 years (45). However, another prospective cohort study found that HI intake from red meat was associated with increased risk of CVD over 6 years in adults aged 45–84 years when the lowest quintile was compared with the highest quintile, but there were no associations for HI intakes from poultry or seafood (44). Future research could further investigate whether the food source in which HI is derived, or consumption of HI with other protective factors in the same meal such as omega-3 in seafood (46) or chlorophyll in green vegetables (47), may attenuate or modify the association between HI intake and MACE. This would allow for better application toward practical recommendations on food group intakes and ideal meal compositions for older adults to reduce risk of MACE.

The different mechanisms of action between different iron intakes could explain the positive association of HI intake with MACE and individual endpoints, and inverse association of NHI intake with all-cause mortality. HI rather than NHI consumption introduces an exogenous haem source to the system which can contribute to haem overload, where circulating levels of haem override the binding capacity of the haem scavenger haemopexin leading to unbound free haem. Free haem can enter cells, catalyse the formation of reactive oxygen species resulting in inflammation and tissue damage (48, 49, 50). Free haem is also pro-inflammatory and impairs vascular function, through attracting leukocytes, platelets, and red blood cells to the vascular endothelium, oxidising low-density lipoprotein and consuming nitric oxide that has many vascular benefits (49, 51). Human hearts with ischaemic cardiomyopathy were cross-sectionally associated with higher haem content, and hearts of mice following coronary ligation also had increased haem content, higher oxidative stress, enhanced cytosis, and worse cardiac function (52). Furthermore, in a cross-sectional study of adults aged 18 years and over, HI intake was positively associated with lipid peroxidation biomarkers, whilst NHI intake was negatively associated indicating the inverse relationships with oxidative damage (53).

There have been different treatments proposed to prevent haem overload. In in vivo research, haemopexin treatment binds free haem in the vascular endothelium and heart which improved cardiac function in mice with haemolytic disorders (54), and reduced infarct volumes in mice following cerebral ischaemia injury (55). Another emerging therapeutic target is the haem-degrading enzyme, haem oxygenase-1, which catalyses the oxidation of haem to generate cardioprotective molecules (56), and may also have a role in preserving nitric oxide bioavailability (51). Previous research has indicated iron restriction as an intervention (48). However, our study found that lower HI intakes were associated with reduced risks of MACE and individual endpoints of MACE in older men, rather than dietary total iron intake. A 1mg/d reduction in HI intake can be achieved through reduced consumption of various food sources such as 65g cooked beef or lamb, or 125g cooked pork or pork products (19). Furthermore, healthy dietary patterns associated with reduced risks of MACE in studies involving older adults already recommend limiting consumption of processed meats and red meats such as the Mediterranean and DASH (Dietary Approaches to Stop Hypertension) dietary patterns (57, 58). Extensive research is required to determine the associations between HI intakes and haem overload, and the maximum threshold of absolute HI intake to avoid contributing to haem overload, which is an area yet to be explored.

There are several study limitations. As our study is observational, causation cannot be determined from cross-sectional analyses on dietary iron intakes with iron status biomarkers, and longitudinal analyses on dietary iron intakes with MACE. For longitudinal analyses, dietary exposure and other measures were only from a single timepoint that may have changed over the follow-up period, possibly resulting in misclassification of exposure factors to some extent but which would be expected to nullify rather than create positive findings. We had a direct measure of total iron intake, but HI and NHI intakes were derived through dietary calculation as HI content of foods is not readily available in nutrient databases. There is interindividual variation in the bioavailability of iron due to different meal compositions with potential enhancers and inhibitors present that were not accounted for (36). We did not account for supplementary iron intakes as detailed data on dosage or levels of supplements were unavailable, and although iron and/or multivitamin supplement use was considered as a covariate, it was not included in the multivariable model derived from backward elimination. Thus, our study conclusions are based on dietary iron intakes and cannot be extrapolated to supplementary iron intakes without further research. Ferritin was not measured to identify iron deficiency and thus could not be included as a covariate. C-reactive protein was not measured, however we adjusted for the available inflammatory biomarker IL-6. Because of small numbers with no participants who experienced an event in a category, some categorical covariates were not included in adjusted models assessing associations between iron intakes with MI and coronary revascularisation. All-cause mortality was included in the composite index of five-point MACE as data on cause-specific mortality was not available. However, we also presented analyses with four-point MACE excluding all-cause mortality. Data on family history of CVD, which could influence CVD risk from attributions of shared genetic and lifestyle factors, was not collected (59). Thus genetic predisposition was not accounted for. However, lifestyle factors were collected and adjusted for in analyses. Furthermore, previous research has shown that family history of CVD as a predictor of CVD risk reduced with increasing age (59) and family history of MI was not a significant predictor of incident MACE in older adults (60). Our study was limited to community-dwelling men who consumed an omnivorous diet, and the results may not apply to older women or those on a plant-based diet.

The strength of our study is that we explored the longitudinal associations between iron intakes with MACE over time. Examining dietary iron intakes as exposure rather than iron status biomarkers, which were not associated with iron intakes in the present study, allows for application as dietary recommendations and practical implementation by individuals to reduce risk of MACE. We used a validated dietitian-administered diet history questionnaire, which has been indicated for older adults due to the non-reliance on short-term memory and low respondent burden (16). We considered multiple confounders for inclusion in the model, and adjustments included NSAID, antiplatelet, anticoagulant, PPI and/or H2RA use that could cause gastrointestinal bleeding, enteropathy and affect iron absorption, energy intake that would also account for under or over-reporting, and individual food groups for the overall dietary pattern consumed. Sensitivity analyses were conducted with the additional adjustment of food subgroups of HI sources. A further strength of CHAMP is that it includes a large and representative group of older Australian men (15).

Importantly, the participants in the present study did not exceed the recommended nutrient reference value upper limit of 45mg per day for total iron intake (61). We did not have ferritin as a measure to identify iron deficiency, however, a sixth of participants were anaemic and almost a quarter had low serum iron. Despite this, per 1mg increment of HI intake was associated with increased risks of five-point MACE, four-point MACE excluding all-cause mortality, all-cause mortality, CCF, and coronary revascularisation. Future research could confirm the mechanisms by which dietary iron intakes influence the development of MACE, and investigate: the effects of different amounts of HI intakes on cardiac function and MACE in randomised controlled trials such as in vivo research in mice; the effects of reducing HI intake whilst maintaining total iron intake on cardiac function and MACE in randomised controlled trials; whether there is a safe level of HI intake; whether the food source in which HI is derived, or consumption of HI with other protective factors in the same meal, or involving an iron deficient population only, or population consuming plant-based diets only, attenuates or modifies the relationships between dietary iron intakes and MACE; and the prospective associations between dietary iron intakes and MACE in populations with a history of MACE, to identify whether HI intake could exacerbate the process and thus the potential role of dietary iron intakes in secondary prevention.

## Conclusion

Higher HI intake was associated with increased risks of five-point MACE, four-point MACE excluding all-cause mortality, all-cause mortality, CCF, and coronary revascularisation in older men aged 75 years and over. Further research is required to confirm the mechanism and investigate the potential of HI restriction for the primary prevention of MACE.

## Funding

The CHAMP study is funded by the National Health and Medical Research Council (project grant no. 301916), Ageing and Alzheimers Research Institute, Ageing and Alzheimers Research Foundation, and the Sydney Medical School Foundation. This research was supported by the Australian Research Council Centre of Excellence in Population Ageing Research (project number CE170100005). RL was supported by an Australian Government Research Training Program Scholarship and an Australian Research Council Centre of Excellence in Population Aging Research Scholarship. RVR was supported by a Charles Perkins Centre Early Career Fellowship from Jennie Mackenzie. The funders were not involved in the conception, design, performance, or approval of this work.

## Electronic Supplementary Material

Supplementary material, approximately 718 KB.
