# Supplementary material for: Haem Iron Intake Is Associated with Increased Major Adverse Cardiovascular Events, All-Cause Mortality, Congestive Cardiac Failure, and Coronary Revascularisation in Older Men: The Concord Health and Ageing in Men Project
Source: J Nutr Health Aging. 2024 Jan 4;27(7):559–70. doi: 10.1007/s12603-023-1945-6 (PMC12929967; doi:10.1007/s12603-023-1945-6)
Supplement: Supplementary file 1 — Supplementary material, approximately 718 KB. [file mmc1.pdf]

I. **Supplementary Table 1.** Associations between different dietary iron intakes (mg/d) and serum iron (μmol/L) using linear regression presented as beta coefficients (95% CI) (n = 522)

II. **Supplementary Table 2.** Associations between different dietary iron intakes and haemoglobin using linear regression presented as beta coefficients (95% CI) (n = 523)

III. **Supplementary Table 3.** Number (%) of major adverse cardiovascular events (MACE) and individual endpoints of MACE events stratified by tertiles of iron intakes (n = 539)

IV. **Supplementary Table 4.** Predictors of five-point MACE in univariate analysis unadjusted using Cox regression presented as hazard ratios (95% CI) (n=539)

V. **Supplementary Table 5.** Associations between dietary iron intakes and individual endpoints of MACE using Cox regression presented as hazard ratios (95% CI) (n = 539)

VI. **Supplementary Table 6.** Associations between dietary iron intakes, MACE and individual endpoints using Cox regression further adjusted with food subgroup intakes presented as hazard ratios (95% CI) (n = 516)

VII. **Supplementary Figure 1.** Linear relationship between HI intake and MACE through plotting decile midpoints of HI intake versus decile beta coefficients from fully adjusted analyses: (A) five-point MACE; (B) four-point MACE excluding all-cause mortality.

VIII. **Supplementary Figure 2.** Survival free of individual endpoints of MACE unadjusted analyses: (A) all-cause mortality; (B) CCF; (C) coronary revascularisation; (D) MI; (E) stroke.

IX. Supplementary Figure 3. Survival free of individual endpoints of MACE fully adjusted analyses: (A) all-cause mortality; (B) CCF; (C) coronary revascularisation; (D) MI; (E) stroke.

Supplementary Table 1. Associations between different dietary iron intakes (mg/d) and serum iron (µmol/L) using linear regression presented as beta coefficients (95% CI) (n = 522)

**Iron intake**		As continuous variable
Total iron[a]		
Model 1		0.04 (-0.02, 0.09) P = .20
Model 2		0.01 (-0.05, 0.07) P = .69
Model 3		
	Without haemoglobin	0.02 (-0.04, 0.08) P = .60
	With haemoglobin	0.02 (-0.04, 0.08) P = .53
Haem iron[b]		
Model 1		-0.25 (-0.77, 0.26) P = .33
Model 2		-0.43 (-1.07, 0.22) P = .19
Model 3		
	Without haemoglobin	-0.48 (-1.14, 0.19) P = .16
	With haemoglobin	-0.57 (-1.19, 0.06) P = .074
Non-haem iron[c]		
Model 1		0.01 (-0.10, 0.13) P = .83
Model 2		-0.08 (-0.23, 0.07) P = .29
Model 3		
	Without haemoglobin	-0.08 (-0.24, 0.08) P = .33
	With haemoglobin	-0.03 (-0.18, 0.12) P = .73

Notes: Model 1 unadjusted (n = 522); Model 2 adjusted by sociodemographic and lifestyle factors (age (continuous), BMI (continuous), country of birth (Australia v. Greece/Italy v. other), marital status (married/de facto v. not married/divorced/separated/widowed/never married/other), age pension (only v. other), alcohol consumption (non-drinker v. ex-drinker v. safe drinker v. harmful drinker), smoking status (non-smoker v. ex-smoker v. current smoker), PASE (continuous), energy intake (continuous), Mediterranean diet score (continuous), number of serves of fruits (continuous), vegetables (continuous), grains (continuous), meat/alternatives (continuous), dairy/alternatives (continuous), and iron and/or multivitamin

supplement use (yes v. no)) (n = 509); Model 3 adjusted by Model 2 plus health (number of medications (continuous), frailty status (robust v. pre-frail v. frail), number of comorbidities (continuous) and IL-6 (continuous)) without haemoglobin (n = 476) and with haemoglobin (continuous) (n = 474)

Supplementary Table 2. Associations between different dietary iron intakes and haemoglobin using linear regression presented as beta coefficients (95% CI) (n = 523)

**Iron intake**	As continuous variable
Total iron[a]	
Model 1	-0.01 (-0.15, 0.12) P = .84
Model 2	0.00 (-0.14, 0.15) P = .96
Model 3	0.00 (-0.14, 0.14) P = .97
Haem iron[b]	
Model 1	0.70 (-0.56, 1.96) P = .27
Model 2	0.72 (-0.82, 2.26) P = .36
Model 3	0.64 (-0.90, 2.17) P = .82
Non-haem iron[c]	
Model 1	-0.13 (-0.41, 0.15) P = .37
Model 2	-0.26 (-0.62, 0.10) P = .16
Model 3	-0.28 (-0.64, 0.08) P = .13

Notes: Model 1 unadjusted (n = 523 for total); Model 2 adjusted by sociodemographic and lifestyle factors (age (continuous), BMI (continuous), country of birth (Australia v. Greece/Italy v. other), marital status (married/de facto v. not married/divorced/separated/widowed/never married/other), age pension (only v. other), alcohol consumption (non-drinker v. ex-drinker v. safe drinker v. harmful drinker), smoking status (non-smoker v. ex-smoker v. current smoker), PASE (continuous), energy intake (continuous), Mediterranean diet score (continuous), number of serves of fruits (continuous), vegetables (continuous), grains (continuous), meat/alternatives (continuous), dairy/alternatives (continuous), and iron and/or multivitamin supplement use (yes v. no)) (n = 509); Model 3 adjusted by Model 2 plus health (number of medications (continuous), frailty status (robust v. pre-frail v. frail), number of comorbidities (continuous) and IL-6 (continuous)) (n = 475 for total)

Supplementary Table 3. Number (%) of major adverse cardiovascular events (MACE) and individual endpoints of MACE events stratified by tertiles of iron intakes (n = 539)

**Iron intake**	Bottom tertile	Middle tertile	Top tertile
Total iron[a]	≤11.26mg/d	11.27-14.75mg/d	≥14.76mg/d
Five-point MACE	63 (35.0)	51 (28.3)	54 (30.2)
Four-point MACE excluding all-cause mortality	38 (39.6)	30 (31.3)	28 (29.2)
All-cause mortality	41 (22.8)	32 (17.8)	38 (21.2)
Myocardial infarction	6 (3.3)	6 (3.3)	8 (4.5)
Congestive cardiac failure	24 (13.3)	20 (11.1)	17 (9.5)
Ischaemic stroke	8 (4.4)	4 (2.2)	5 (2.8)
Coronary revascularisation	3 (1.7)	7 (3.9)	5 (2.8)
Haem iron[b]	≤1.40mg/d	1.41-2.10mg/d	≥2.11mg/d
Five-point MACE	56 (31.1)	59 (32.8)	53 (29.6)
Four-point MACE excluding all-cause mortality	31 (32.3)	33 (34.4)	32 (33.3)
All-cause mortality	35 (19.4)	40 (22.2)	36 (20.1)
Myocardial infarction	9 (5.0)	7 (3.9)	4 (2.2)
Congestive cardiac failure	16 (8.9)	25 (13.9)	20 (11.2)
Ischaemic stroke	7 (3.9)	5 (2.8)	5 (2.8)
Coronary revascularisation	3 (1.7)	3 (1.7)	9 (5.0)
Non-haem iron[c]	≤9.42mg/d	9.43-12.77mg/d	≥12.78mg/d
Five-point MACE	64 (35.6)	47 (26.1)	57 (31.8)
Four-point MACE excluding all-cause mortality	39 (40.6)	29 (30.2)	28 (29.2)
All-cause mortality	42 (23.3)	29 (16.1)	40 (22.3)
Myocardial infarction	6 (3.3)	7 (3.9)	7 (3.9)
Congestive cardiac failure	24 (13.3)	21 (11.7)	16 (8.9)
Ischaemic stroke	8 (4.4)	4 (2.2)	5 (2.8)
Coronary revascularisation	6 (3.3)	3 (1.7)	6 (3.4)

[a] Bottom tertile ≤11.26mg/d, n = 180 with median (IQR) 9.59 (8.24, 10.41); middle tertile 11.27-14.75mg/d, n = 180 with median (IQR) 12.71 (11.92, 13.66); top tertile ≥14.76mg/d, n = 179 with median (IQR) 17.64 (15.98, 20.13)

[b] Bottom tertile ≤1.40mg/d, n = 180 with median (IQR) 1.00 (0.75, 1.21); middle tertile 1.41-2.10mg/d, n = 180 with median (IQR) 1.74 (1.58, 1.92); top tertile ≥2.11mg/d, n = 179 with median (IQR) 2.65 (2.38, 3.14)

[c] Bottom tertile ≤9.42mg/d, n = 180 with median (IQR) 7.93 (6.72, 8.75); middle tertile 9.43-12.77mg/d, n = 180 with median (IQR) 10.86 (10.15, 11.62); top tertile ≥12.78mg/d, n = 179, with median (IQR) 15.30 (13.81, 17.55)

Supplementary Table 4. Predictors of five-point MACE in univariate analysis unadjusted using Cox regression presented as hazard ratios (95% CI) (n=539)

Risk factor	Hazard ratios (95% CI)
Age	1.11 (1.08, 1.14) P < .001
BMI (kg/m^2) (n = 533)	0.96 (0.92, 0.99) P = .012
Country of birth	
Australia (reference)	1
Greece/ Italy	0.68 (0.46, 1.02) P = .061
Other	0.88 (0.61, 1.28) P = .51
Source of income (n = 538)	
Age Pension only	1
Other	0.78 (0.57, 1.05) P = .11
Energy intake	1.00 (1.00,1.00) P = .61
Vegetables	0.93 (0.86, 1.01) P = .067
Fruit	0.94 (0.84, 1.05) P = .25
Meat/alternatives	0.81 (0.71, 0.93) P = .002
Grains	0.93 (0.86, 1.00) P = .040
Medication use	
Neither NSAID, anticoagulant, antiplatelet, PPI and/or H2RA (reference)	1
NSAID, anticoagulant and/or antiplatelet only	1.32 (0.92, 1.90) P = .14
PPI and/or H2RA only	1.23 (0.76, 2.00) P = .40
NSAID, anticoagulant and/or antiplatelet with PPI and/or H2RA	1.76 (1.12, 2.76) P = .0.13
Haemoglobin (g/L) (n = 523)	0.98 (0.97, 0.99) P < .001
Anaemia (n = 523) (Haemoglobin<130g/L)	
No (reference)	1
Yes	1.65 (1.13, 2.41) P = .009
Frailty status (n = 534)	
Robust (reference)	1
Pre-frail	1.59 (1.14, 2.20) P = .006
Frail	5.47 (3.19, 9.38) P < .001

Chronic Kidney Disease (eGFR <60mL/min/1.73m^2) (n = 525)		
	No (reference)	1
	Yes	1.50 (1.10, 2.06) P = .011

Supplementary Table 5. Associations between dietary iron intakes and individual endpoints of MACE using Cox regression presented as hazard ratios (95% CI) (n = 539)

**Iron intake**	Bottom tertile (reference category)	Middle tertile	Top tertile	As continuous variable
Total iron[a]	≤11.26mg/d	11.27-14.75mg/d	≥14.76mg/d	+1mg/d
**All-cause mortality**				
Model 1	1	0.68 (0.43, 1.08) P = .10	0.81 (0.52, 1.26) P = .36	1.01 (0.99, 1.03) P = .24
Model 2	1	0.63 (0.38, 1.04) P = .071	0.63 (0.35, 1.13) P = .12	1.00 (0.98, 1.02) P = .86
Model 3	1	0.63 (0.37, 1.06) P = .084	0.63 (0.35, 1.13) P = .12	1.00 (0.98, 1.02) P = .83
**Congestive cardiac failure**				
Model 1	1	0.75 (0.41, 1.36) P = .34	0.64 (0.34, 1.19) P = .16	0.99 (0.95, 1.03) P = .62
Model 2	1	1.02 (0.52, 2.00) P = .95	1.06 (0.47, 2.39) P = .88	1.00 (0.97, 1.03) P = .99
Model 3	1	1.11 (0.56, 2.21) P = .77	0.99 (0.43, 2.25) P = .98	1.00 (0.96, 1.03) P = 1.00
**Coronary revascularisation**				
Model 1	1	2.09 (0.54, 8.11) P = .29	1.54 (0.37, 6.47) P = .56	1.00 (0.94, 1.06) P = .99
Model 2	1	2.56 (0.63, 10.47) P = .19	1.70 (0.30, 9.85) P = .55	1.00 (0.93, 1.08) P = .97
Model 3[†]	1	2.44 (0.59, 10.15) P = .22	1.66 (0.28, 9.69) P = .58	1.00 (0.92, 1.08) P = .98

**Myocardial infarction**				
Model 1	1	0.88 (0.28, 2.73)	1.15 (0.40, 3.34)	1.01 (0.97, 1.05)
		P = .82	P = .79	P = .59
Model 2	1	1.26 (0.37, 4.30)	1.76 (0.45, 6.80)	1.00 (0.96, 1.05)
		P = .72	P = .42	P = .84
Model 3[#]	1	1.55 (0.40, 5.98)	2.20 (0.49, 9.87)	1.01 (0.97, 1.06)
		P = .52	P = .31	P = .67
**Ischaemic stroke**				
Model 1	1	0.45 (0.14, 1.51)	0.57 (0.19, 1.75)	0.99 (0.93, 1.06)
		P = .20	P = .33	P = .85
Model 2	1	0.61 (0.17, 2.23)	0.94 (0.22, 4.13)	1.01 (0.96, 1.06)
		P = .45	P = .94	P = .71
Model 3	1	0.59 (0.16, 2.22)	0.78 (0.17, 3.56)	1.01 (0.95, 1.06)
		P = .43	P = .74	P = .83
Haem iron[b]	≤1.40mg/d	1.41-2.10mg/d	≥2.11mg/d	+1mg/d
**All-cause mortality**				
Model 1	1	1.16 (0.74, 1.83)	1.03 (0.65, 1.64)	1.11 (0.93, 1.33)
		P = .52	P = .90	P = .23
Model 2	1	1.39 (0.83, 2.33)	1.49 (0.82, 2.71)	1.50 (1.14, 1.97)
		P = .22	P = .19	P = .004
Model 3	1	1.56 (0.91, 2.67)	1.60 (0.87, 2.95)	1.51 (1.15, 1.99)
		P = .10	P = .13	P = .003
**Congestive cardiac failure**				
Model 1	1	1.61 (0.86, 3.01)	1.25 (0.65, 2.41)	1.14 (0.89, 1.47)
		P = .14	P = .51	P = .30
Model 2	1	2.70 (1.32, 5.55)	2.92 (1.27, 6.76)	2.01 (1.41, 2.87)
		P = .007	P = .012	P < .001
Model 3	1	3.10 (1.43, 6.73)	3.07 (1.28, 7.35)	2.08 (1.45, 2.98)
		P = .004	P = .012	P < .001

**Coronary revascularisation**				
Model 1	1	0.99 (0.20, 4.89) P = .99	3.04 (0.82, 11.22) P = .096	1.88 (1.30, 2.72) P = .001
Model 2	1	0.68 (0.13, 3.56) P = .65	2.11 (0.49, 9.16) P = .32	1.98 (1.20, 3.26) P = .007
Model 3[†]	1	0.70 (0.13, 3.81) P = .68	2.13 (0.49, 9.36) P = .32	1.89 (1.15, 3.10) P = .012
**Myocardial infarction**				
Model 1	1	0.78 (0.29, 2.11) P = .63	0.44 (0.14, 1.43) P = .17	0.78 (0.46, 1.32) P = .35
Model 2	1	0.90 (0.30, 2.71) P = .85	0.58 (0.15, 2.35) P = .45	1.01 (0.51, 1.98) P = .98
Model 3[#]	1	0.89 (0.27, 2.95) P = .85	0.58 (0.12, 2.81) P = .50	1.09 (0.56, 2.14) P = .80
**Ischaemic stroke**				
Model 1	1	0.71 (0.23, 2.25) P = .56	0.72 (0.23, 2.25) P = .57	0.90 (0.53, 1.55) P = .70
Model 2	1	0.69 (0.20, 2.44) P = .57	0.83 (0.21, 3.34) P = .79	1.08 (0.53, 2.21) P = .84
Model 3	1	0.67 (0.19, 2.35) P = .53	0.81 (0.20, 3.31) P = .76	1.06 (0.53, 2.13) P = .87
Non-haem iron[c]	≤9.42mg/d	9.43-12.77mg/d	≥12.78mg/d	+1mg/d
**All-cause mortality**				
Model 1	1	0.60 (0.38, 0.97) P = .037	0.84 (0.54, 1.29) P = .42	0.99 (0.95, 1.03) P = .57
Model 2	1	0.54 (0.32, 0.90) P = .018	0.65 (0.36, 1.17) P = .15	0.95 (0.89, 1.01) P = .12
Model 3	1	0.56 (0.33, 0.96) P = .035	0.65 (0.35, 1.18) P = .16	0.94 (0.88, 1.00) P = .055

**Congestive cardiac failure**				
Model 1	1	0.79 (0.44, 1.43)<br>P = .44	0.60 (0.32, 1.13)<br>P = .11	0.97 (0.90, 1.03)<br>P = .31
Model 2	1	1.02 (0.53, 1.99)<br>P = .94	0.86 (0.37, 1.99)<br>P = .73	1.02 (0.94, 1.10)<br>P = .68
Model 3	1	1.11 (0.56, 2.21)<br>P = .77	0.84 (0.35, 1.99)<br>P = .69	1.00 (0.92, 1.08)<br>P = .95
**Coronary revascularisation**				
Model 1	1	0.44 (0.11, 1.76)<br>P = .24	0.91 (0.29, 2.84)<br>P = .87	1.00 (0.88, 1.13)<br>P = .99
Model 2	1	0.53 (0.12, 2.27)<br>P = .39	0.94 (0.21, 4.24)<br>P = .93	1.00 (0.84, 1.19)<br>P = .99
Model 3[†]	1	0.45 (0.10, 1.97)<br>P = .29	0.84 (0.18, 3.98)<br>P = .82	1.00 (0.83, 1.20)<br>P = .97
**Myocardial infarction**				
Model 1	1	1.03 (0.34, 3.06)<br>P = .96	1.01 (0.34, 3.01)<br>P = .99	1.03 (0.95, 1.13)<br>P = .45
Model 2	1	1.28 (0.40, 4.12)<br>P = .68	1.31 (0.32, 5.40)<br>P = .71	1.03 (0.94, 1.13)<br>P = .56
Model 3[#]	1	1.52 (0.43, 5.36)<br>P = .52	1.48 (0.32, 6.90)<br>P = .62	1.04 (0.93, 1.15)<br>P = .52
**Ischaemic stroke**				
Model 1	1	0.46 (0.14, 1.53)<br>P = .20	0.57 (0.19, 1.76)<br>P = .33	0.93 (0.81, 1.06)<br>P = .28
Model 2	1	0.62 (0.17, 2.30)<br>P = .48	0.95 (0.21, 4.25)<br>P = .94	0.99 (0.83, 1.18)<br>P = .92
Model 3	1	0.65 (0.17, 2.47)<br>P = .53	0.93 (0.18, 3.85)<br>P = .82	0.96 (0.81, 1.14)<br>P = .65

Notes: Model 1 unadjusted (n = 539 for total, 111 all-cause mortality, 15 coronary revascularisation, 61 congestive cardiac failure, 20 myocardial infarction, and 17 stroke); Model 2 adjusted by age (continuous), BMI (continuous), country of birth (Australia v. Greece/Italy v. other), age pension (only v. other), energy intake (continuous), number of serves of vegetables (continuous), fruit (continuous), meat/alternatives (continuous), grains (continuous) (n = 532 for total, 108 all-cause mortality, 60 congestive cardiac failure, 15 coronary revascularisation, 20 myocardial infarction, and 17 stroke); Model 3 adjusted by Model 2 plus NSAID, anticoagulant, antiplatelet and/or PPI or H2RA use (NSAID, anticoagulant and/or antiplatelet only v. PPI and/or H2RA only v. NSAID, anticoagulant and/or antiplatelet with PPI and/or H2RA v. neither NSAID, antiplatelet, anticoagulant, PPI or H2RA), haemoglobin (continuous), frailty status (robust v. pre-frail v. frail), and CKD (yes v. no) (n = 516 for total, 106 all-cause mortality, 57 congestive cardiac failure, and 17 stroke).

[†]Due to small numbers frailty status could not be included as covariates for coronary revascularisation. Model 3 (n = 516 for total and 15 coronary revascularisation).

[#]Due to small numbers NSAID, anticoagulant, antiplatelet, PPI and/or H2RA use could not be included as a covariate for myocardial infarction. Model 3 (n = 516 for total and 18 myocardial infarction).

[a] Bottom tertile ≤11.26mg/d, n = 180 with median (IQR) 9.59 (8.24, 10.41); middle tertile 11.27-14.75mg/d, n = 180 with median (IQR) 12.71 (11.92, 13.66); top tertile ≥14.76mg/d, n = 179 with median (IQR) 17.64 (15.98, 20.13)

[b] Bottom tertile ≤1.40mg/d, n = 180 with median (IQR) 1.00 (0.75, 1.21); middle tertile 1.41-2.10mg/d, n = 180 with median (IQR) 1.74 (1.58, 1.92); top tertile ≥2.11mg/d, n = 179 with median (IQR) 2.65 (2.38, 3.14)

[c] Bottom tertile ≤9.42mg/d, n = 180 with median (IQR) 7.93 (6.72, 8.75); middle tertile 9.43-12.77mg/d, n = 180 with median (IQR) 10.86 (10.15, 11.62); top tertile ≥12.78mg/d, n = 179, with median (IQR) 15.30 (13.81, 17.55)

Supplementary Table 6. Associations between dietary iron intakes, MACE and individual endpoints using Cox regression further adjusted with food subgroup intakes presented as hazard ratios (95% CI) (n = 516)

**Iron intake**	Bottom tertile (reference category)	Middle tertile	Top tertile	As continuous variable
Total iron[a]	≤11.26mg/d	11.27-14.75mg/d	≥14.76mg/d	+1mg/d
Five-point MACE	1	0.79 (0.52, 1.20) P = .27	0.82 (0.50, 1.34) P = .43	1.00 (0.99, 1.02) P = .74
Four-point MACE excluding all-cause mortality		0.97 (0.56, 1.67) P = .90	1.09 (0.57, 2.09) P = .80	1.00 (0.97, 1.02) P = .93
All-cause mortality	1	0.61 (0.36, 1.03) P = .065	0.63 (0.34, 1.17) P = .14	1.00 (0.98, 1.02) P = .99
Congestive cardiac failure	1	1.08 (0.54, 2.15) P = .83	1.07 (0.46, 2.50) P = .87	0.99 (0.96, 1.02) P = .59
Coronary revascularisation[†]	1	1.78 (0.42, 7.62) P = .43	1.37 (0.23, 8.29) P = .73	1.00 (0.91, 1.09) P = .96
Myocardial infarction[#]	1	1.42 (0.36, 5.54) P = .62	2.27 (0.47, 10.88) P = .31	1.01 (0.97, 1.05) P = .71
Ischaemic stroke	1	0.57 (0.15, 2.18) P = .42	0.81 (0.17, 3.82) P = .79	1.00 (0.94, 1.06) P = .95
Haem iron[b]	≤1.40mg/d	1.41-2.10mg/d	≥2.11mg/d	+1mg/d
Five-point MACE	1	1.20 (0.75, 1.91) P = .45	1.09 (0.60, 2.00) P = .78	1.42 (1.05, 1.91) P = .024
Four-point MACE excluding all-cause mortality		1.17 (0.63, 2.18) P = .62	1.14 (0.51, 2.53) P = .75	1.53 (1.08, 2.17) P = .016
All-cause mortality	1	1.35 (0.75, 2.40) P = .32	1.22 (0.58, 2.56) P = .61	1.51 (1.03, 2.22) P = .036

Congestive cardiac failure	1	2.88 (1.26, 6.60) P = .013	2.66 (0.92, 7.66) P = .070	2.10 (1.40, 3.16) P < .001
Coronary revascularisation[†]	1	0.59 (0.10, 3.49) P = .56	1.34 (0.20, 8.91) P = .76	2.80 (1.18, 6.60) P = .019
Myocardial infarction[#]	1	0.43 (0.11, 1.68) P = .22	0.14 (0.01, 1.28) P = .081	0.58 (0.19, 1.75) P = .34
Ischaemic stroke	1	0.47 (0.12, 1.89) P = .29	0.41 (0.06, 2.67) P = .35	0.72 (0.24, 2.13) P = .55
Non-haem iron[c]	≤9.42mg/d	9.43-12.77mg/d	≥12.78mg/d	+1mg/d
Five-point MACE	1	0.75 (0.49, 1.15) P = .19	0.91 (0.55, 1.50) P = .71	0.98 (0.94, 1.03) P = .40
Four-point MACE excluding all-cause mortality		0.98 (0.56, 1.70) P = .94	1.12 (0.57, 2.22) P = .75	1.02 (0.96, 1.08) P = .60
All-cause mortality	1	0.57 (0.33,0.98) P = .043	0.70 (0.37, 1.33) P = .28	0.94 (0.88, 1.01) P = .071
Congestive cardiac failure	1	1.24 (0.62, 2.49) P = .54	1.01 (0.41, 2.49) P = .99	1.01 (0.93, 1.09) P = .90
Coronary revascularisation[†]	1	0.54 (0.12, 2.47) P = .43	1.16 (0.23, 5.89) P = .86	1.01 (0.84, 1.23) P = .90
Myocardial infarction[#]	1	1.64 (0.44, 6.04) P = .46	1.76 (0.33, 9.27) P = .50	1.03 (0.92, 1.14) P = .65
Ischaemic stroke	1	0.69 (0.18, 2.68) P = .59	0.95 (0.19, 4.79) P = .95	0.96 (0.81, 1.13) P = .59

Notes: Adjusted for age (continuous), BMI (continuous), country of birth (Australia v. Greece/Italy v. other), age pension (only v. other), energy intake (continuous), number of serves of vegetables (continuous), fruit (continuous), meat/alternatives (continuous), grains (continuous), NSAID, anticoagulant, antiplatelet and/or PPI or H2RA use (NSAID, anticoagulant and/or antiplatelet only v. PPI and/or H2RA only v. NSAID, anticoagulant and/or antiplatelet with PPI and/or H2RA v. neither NSAID, antiplatelet, anticoagulant, PPI or H2RA), haemoglobin (continuous), frailty status (robust v. pre-frail v. frail), and CKD (yes v.

no), number of serves of red meat (continuous), poultry (continuous), processed meat (continuous), and seafood (continuous) (n = 516 for total, 160 five-point MACE, 92 four-point MACE excluding all-cause mortality, 106 all-cause mortality, 57 congestive cardiac failure and 17 stroke).

[†]Due to small numbers frailty status could not be included as covariates for coronary revascularisation (n = 516 for total and 15 coronary revascularisation).

[#]Due to small numbers NSAID, anticoagulant, antiplatelet, PPI and/or H2RA use could not be included as a covariate for myocardial infarction (n = 516 for total and 18 for myocardial infarction).

[a] Bottom tertile ≤11.26mg/d, n = 180 with median (IQR) 9.59 (8.24, 10.41); middle tertile 11.27-14.75mg/d, n = 180 with median (IQR) 12.71 (11.92, 13.66); top tertile ≥14.76mg/d, n = 179 with median (IQR) 17.64 (15.98, 20.13)

[b] Bottom tertile ≤1.40mg/d, n = 180 with median (IQR) 1.00 (0.75, 1.21); middle tertile 1.41-2.10mg/d, n = 180 with median (IQR) 1.74 (1.58, 1.92); top tertile ≥2.11mg/d, n = 179 with median (IQR) 2.65 (2.38, 3.14)

[c] Bottom tertile ≤9.42mg/d, n = 180 with median (IQR) 7.93 (6.72, 8.75); middle tertile 9.43-12.77mg/d, n = 180 with median (IQR) 10.86 (10.15, 11.62); top tertile ≥12.78mg/d, n = 179, with median (IQR) 15.30 (13.81, 17.55)

Supplementary Figure 1. Linear relationship between HI intake and MACE through plotting decile midpoints of HI intake versus decile beta coefficients from fully adjusted analyses: (A) five-point MACE; (B) four-point MACE excluding all-cause mortality.

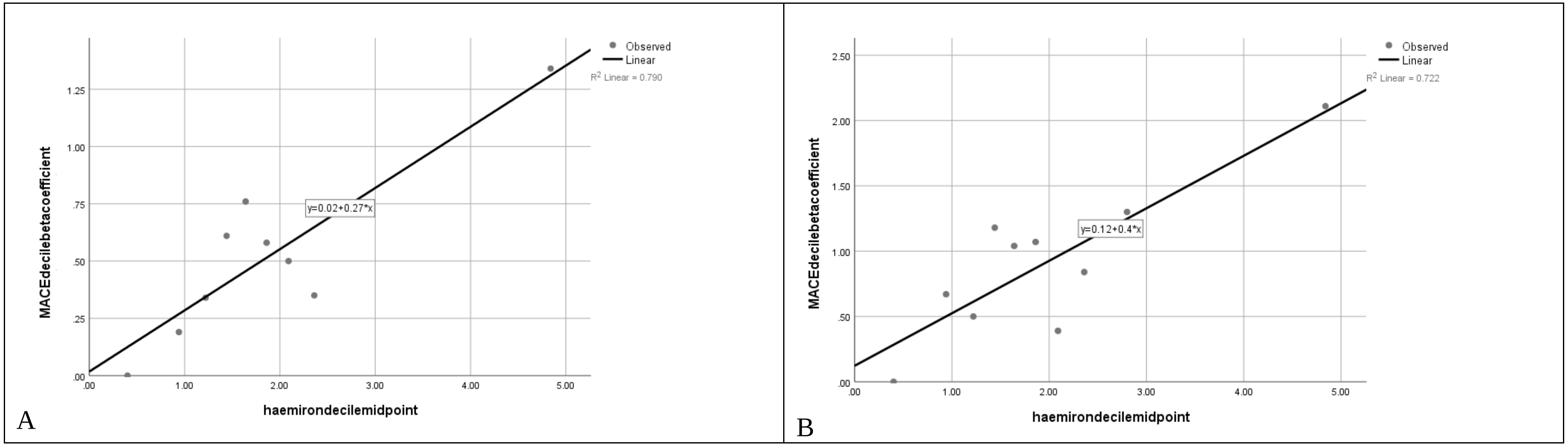

Supplementary Figure 2. Survival free of individual endpoints of MACE unadjusted analyses: (A) all-cause mortality; (B) CCF; (C) coronary revascularisation; (D) MI; (E) stroke. MACE = major adverse cardiovascular event; CCF = congestive cardiac failure; revas = coronary revascularisation; MI = myocardial infarction; Cum = cumulative; Futime = follow-up time.

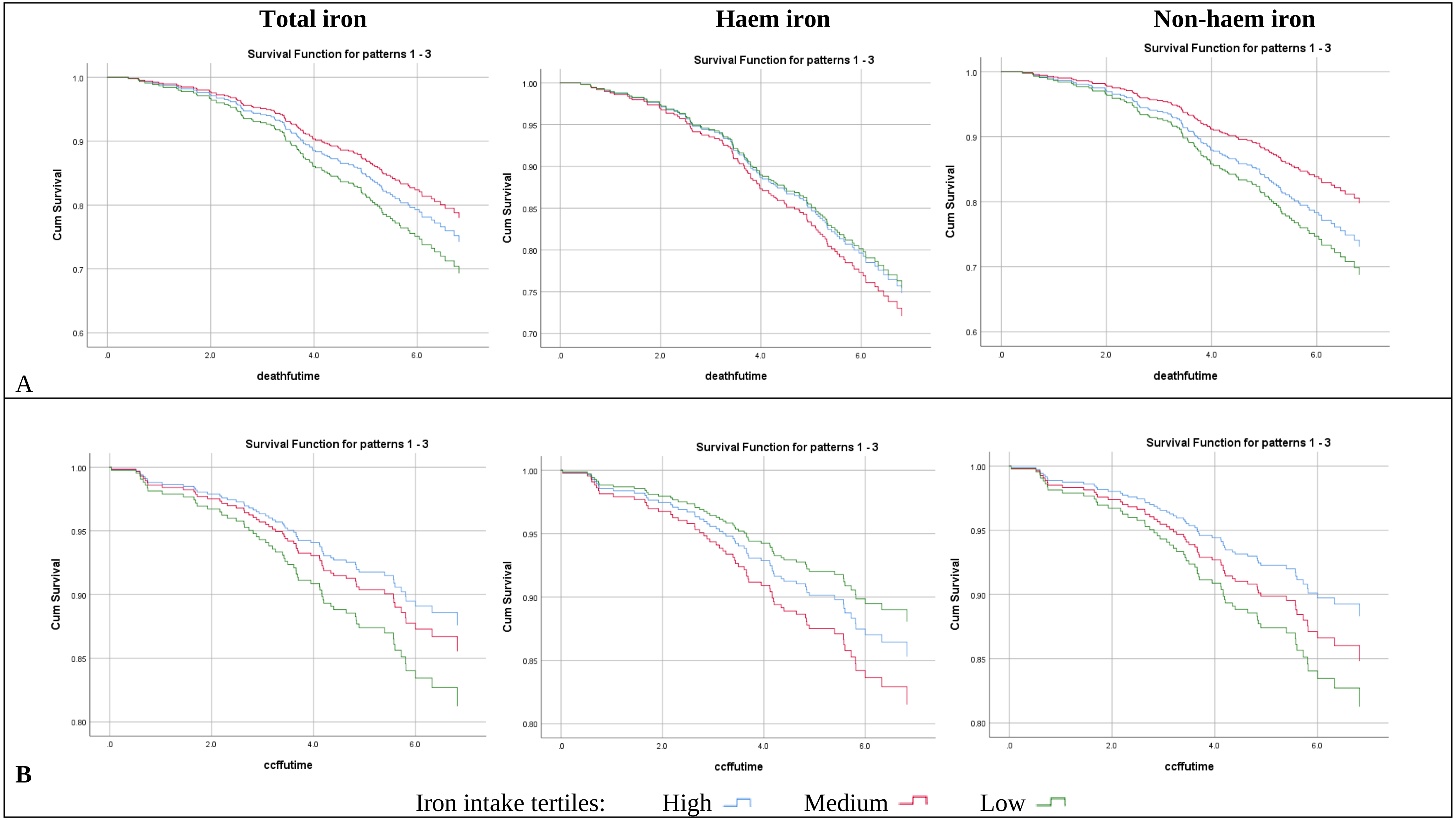

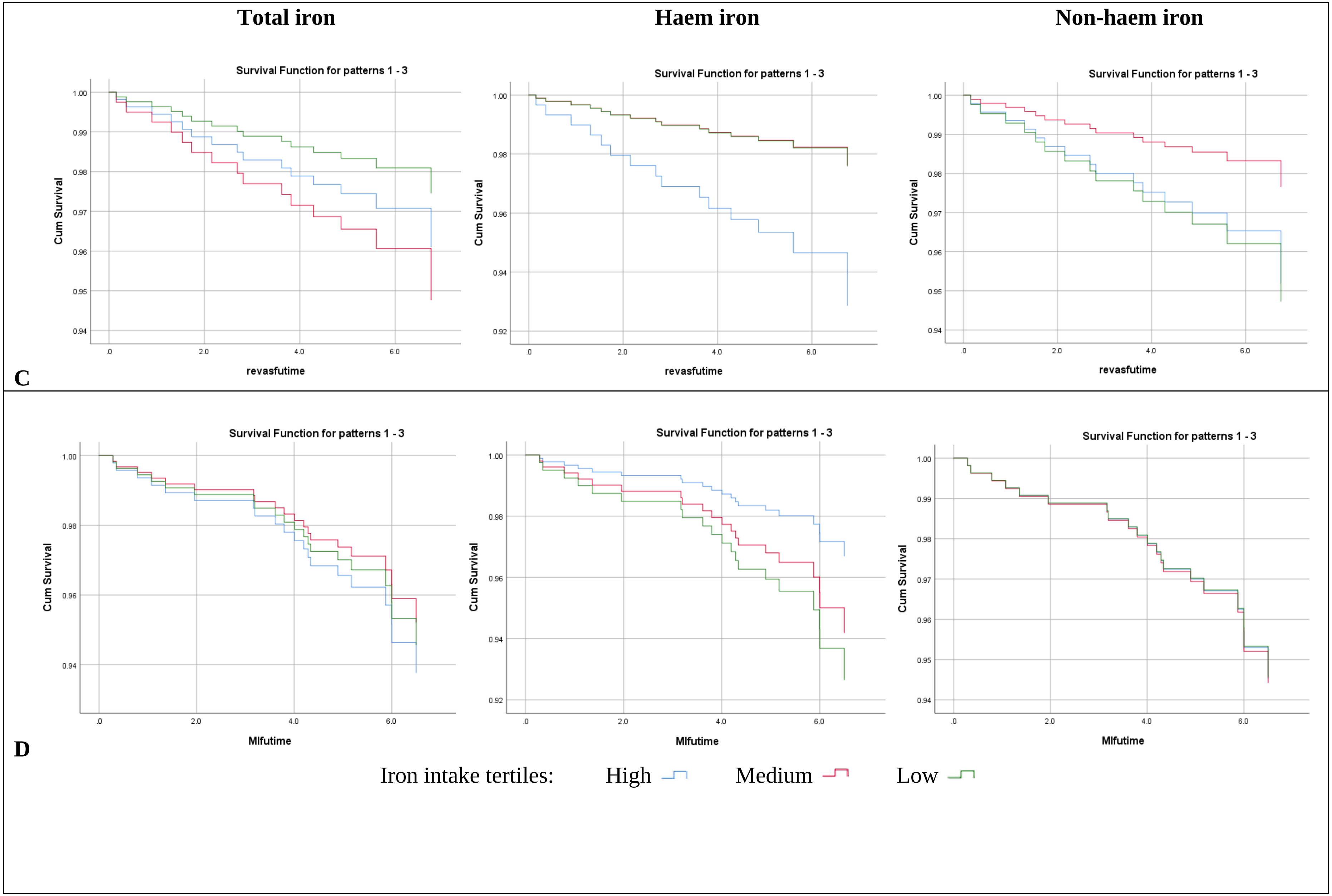
Total iron
Haem iron
Non-haem iron
Survival Function for patterns 1 - 3
Cum Survival
revasfutime
C
MIfutime
D
Iron intake tertiles: High Medium Low

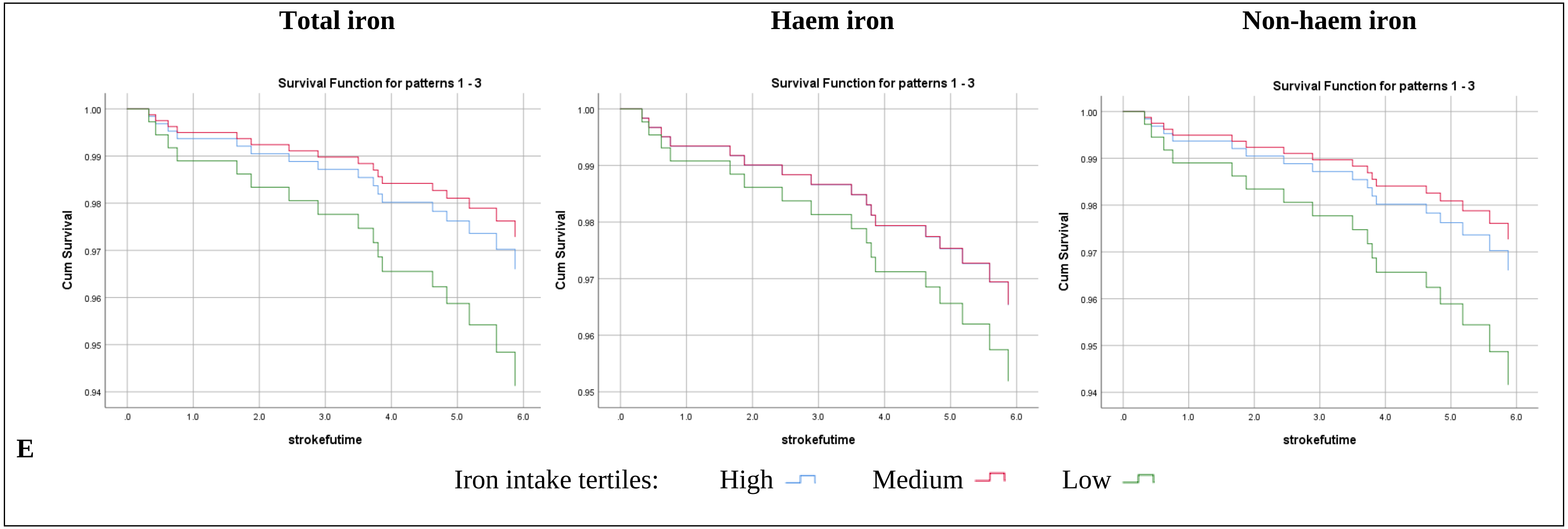
Total iron
Haem iron
Non-haem iron
Survival Function for patterns 1 - 3
Cum Survival
1.00
0.99
0.98
0.97
0.96
0.95
0.94
.0
1.0
2.0
3.0
4.0
5.0
6.0
strokefutime
Iron intake tertiles:
High
Medium
Low

E

Supplementary Figure 3. Survival free of individual endpoints of MACE fully adjusted analyses: (A) all-cause mortality; (B) CCF; (C) coronary revascularisation; (D) MI; (E) stroke. MACE = major adverse cardiovascular event; CCF = congestive cardiac failure; revas = coronary revascularisation; MI = myocardial infarction; Cum = cumulative; Futime = follow-up time.

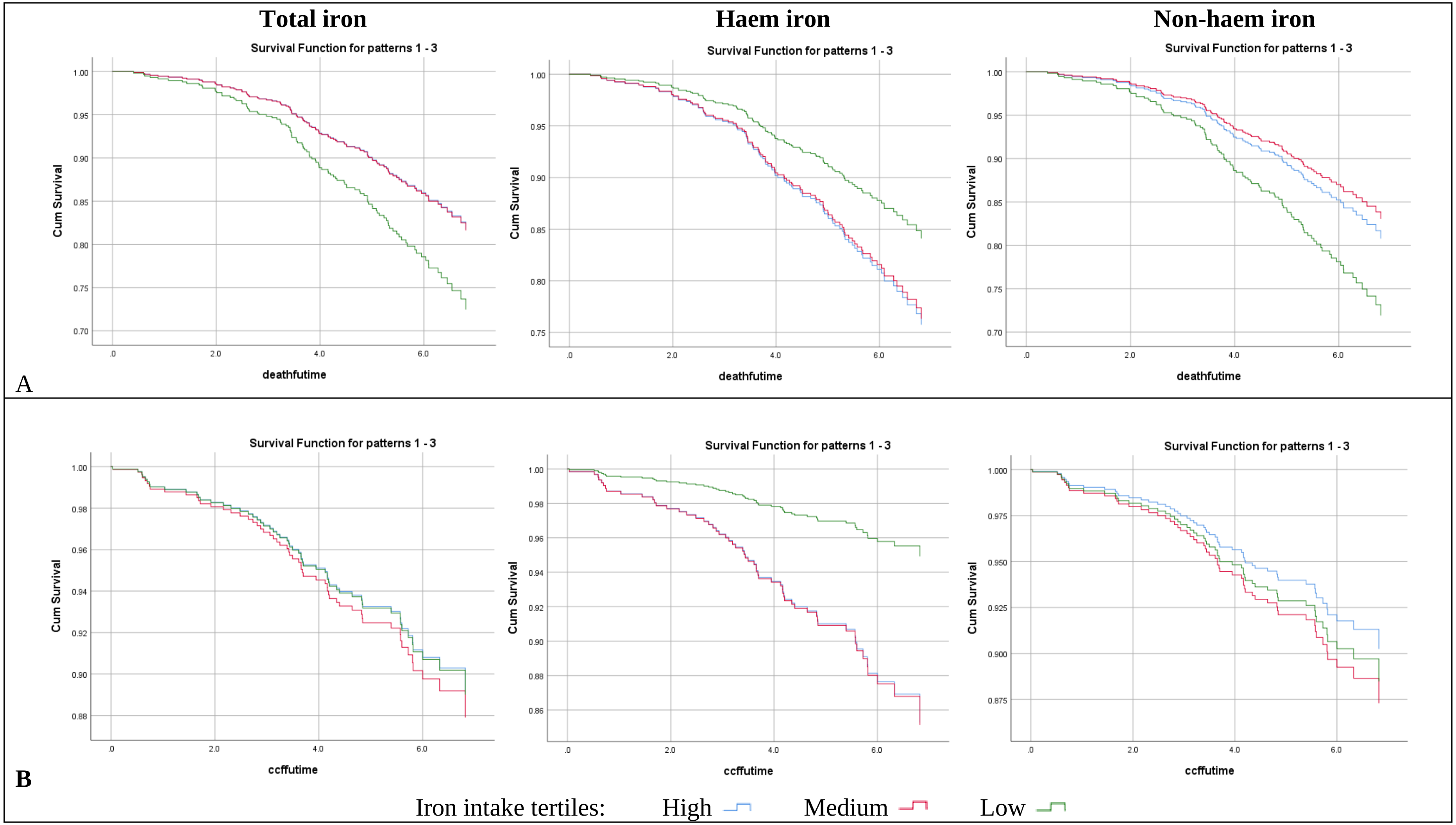

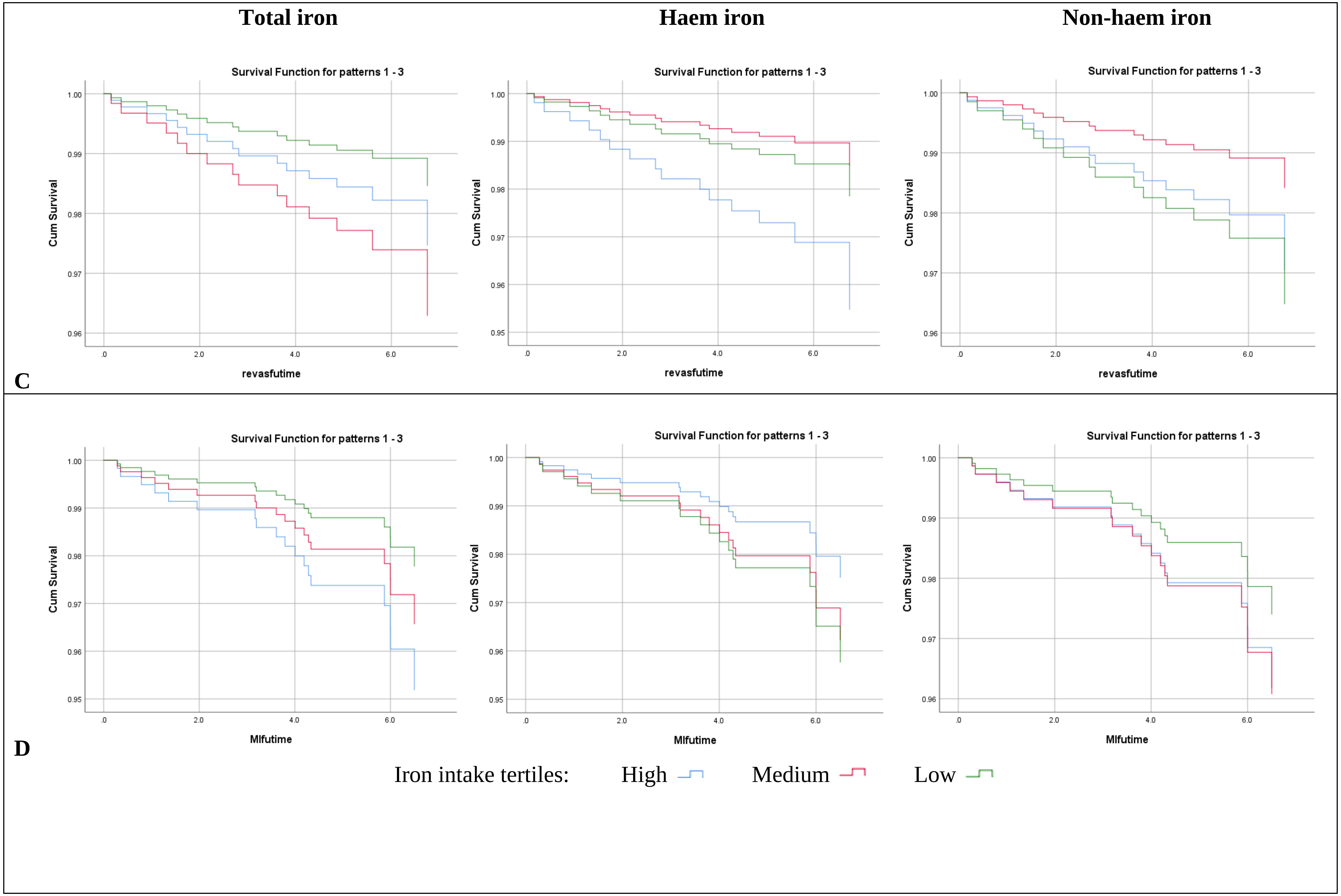
Total iron
Haem iron
Non-haem iron
Survival Function for patterns 1 - 3
Cum Survival
revasfutime
C
MIfutime
D
Iron intake tertiles: High Medium Low

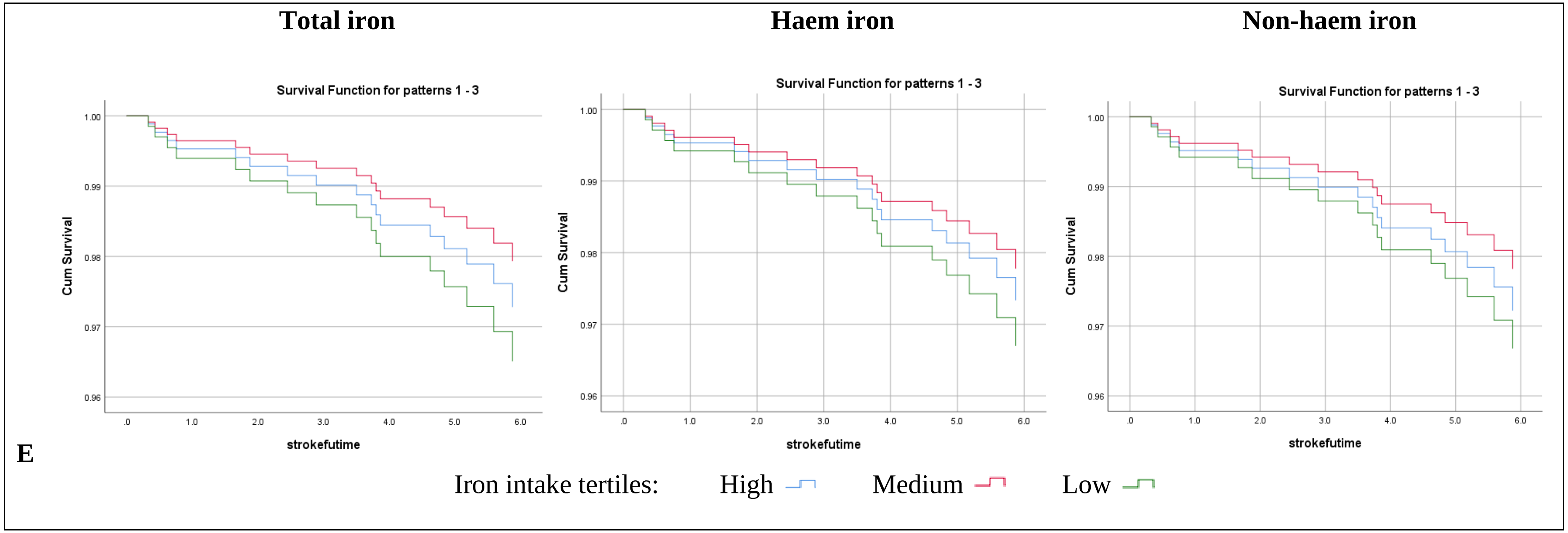
Total iron
Haem iron
Non-haem iron
Survival Function for patterns 1 - 3
Cum Survival
strokefutime
1.00
0.99
0.98
0.97
0.96
.0
1.0
2.0
3.0
4.0
5.0
6.0
E
Iron intake tertiles:
High
Medium
Low
